# A review on biofiltration techniques: recent advancements in the removal of volatile organic compounds and heavy metals in the treatment of polluted water

**DOI:** 10.1080/21655979.2022.2050538

**Published:** 2022-03-28

**Authors:** Rekha Pachaiappan, Lorena Cornejo-Ponce, Rathika Rajendran, Kovendhan Manavalan, Vincent Femilaa Rajan, Fathi Awad

**Affiliations:** a Departamento de Ingeniería Mecánica, Facultad de Ingeniería, Universidad de Tarapacá, Arica, Chile; b Department of Physics, A.D.M. College for Women (Autonomous), Nagapattinam, 611001, India; c Department of Physics and Nanotechnology, SRM Institute of Science and Technology, Kattankulathur, India; d Department of Sustainable Energy Management, Stella Maris College (Autonomous), Chennai - 600086, India; e Department of Allied Health Professionals, Faculty of Medical and Health Sciences, Liwa College of Technology, Abu Dhabi, UAE

**Keywords:** Biological method, biofilters, volatile organic compounds, heavy metals, biodegradation, pollutants, wastewater treatment

## Abstract

Good quality of water determines the healthy life of living beings on this earth. The cleanliness of water was interrupted by the pollutants emerging out of several human activities. Industrialization, urbanization, heavy population, and improper disposal of wastes are found to be the major reasons for the contamination of water. Globally, the inclusion of volatile organic compounds (VOCs) and heavy metals released by manufacturing industries, pharmaceuticals, and petrochemical processes have created environmental issues. The toxic nature of these pollutants has led researchers, scientists, and industries to exhibit concern toward the complete eradication of them. In this scenario, the development of wastewater treatment methodologies at low cost and in an eco-friendly way had gained importance at the international level. Recently, bio-based technologies were considered for environmental remedies. Biofiltration-based works have shown a significant result for the removal of volatile organic compounds and heavy metals in the treatment of wastewater. This was done with several biological sources such as bacteria, fungi, algae, plants, yeasts, etc. The biofiltration technique is cost-effective, simple, biocompatible, sustainable, and eco-friendly compared to conventional techniques. This review article provides deep insight into biofiltration technologies engaged in the removal of volatile organic compounds and heavy metals in the wastewater treatment process.

## Graphical abstract

**Figure:**
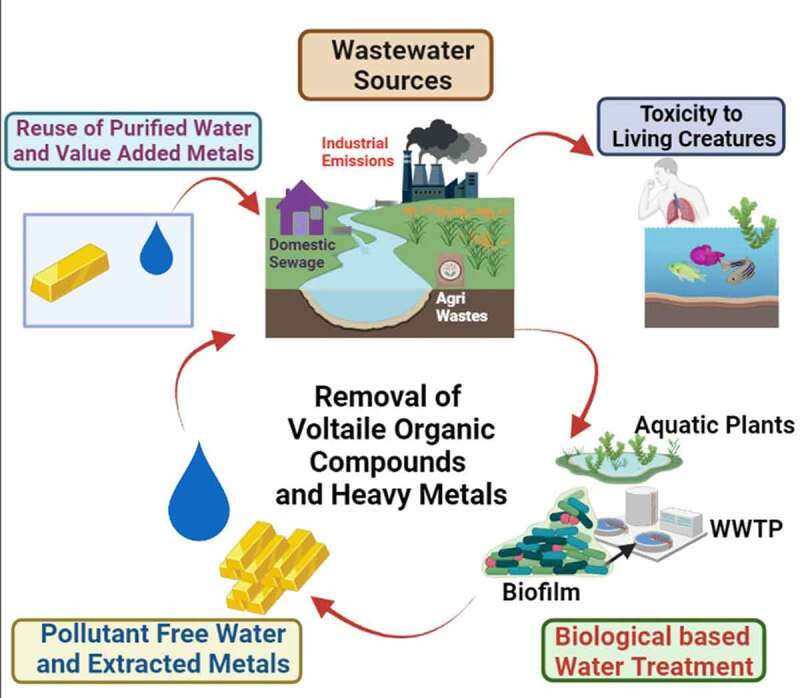

## 1. Introduction

Water pollution has become a major threat to human health and the environment. In 2015, the United Nations had given out ‘The Sustainable Development Goals (SDGs)’, also known as Global Goals. There are 17 goals that have to be implemented to protect our planet earth, remove poverty, and ensure global prosperity and peace by the year 2030 [1]. The sixth goal represents sustainable water management to provide potable water and good sanitation for people. The rise in population, urbanization, and industrialization are responsible for water pollution. Though water is found abundantly on the earth, only 3% of water is potable, whereas the remaining 97% is present as salty water in oceans. In this scenario, 3% of water was contaminated by biological agents, chemicals, and radioactive elements ejected from improper disposal of wastes about 80% into water sources [2]. It was estimated that 2 billion people utilize contaminated water, leading to water-borne diseases. Worldwide, 485,000 deaths occur from diarrheal disease due to polluted water. While considering pollutants, volatile organic compounds (VOCs) and heavy metals are important pollutants. Some are classified as carcinogens and toxic components causing environmental deterioration along with health hazards to living beings. Hence, the demand occurs for the emergence of technology in rectification or restoring the natural water resources to receive a healthy living.

**Figure 1.**
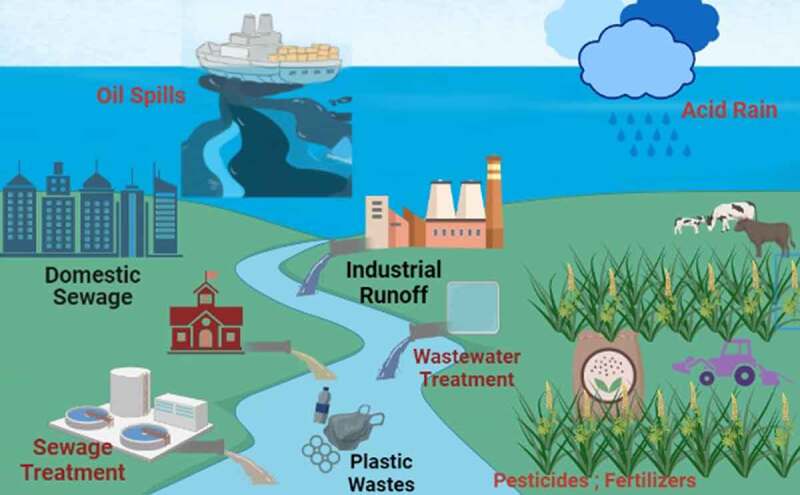
Schematic illustration representing water resource contamination by various pollutants from urban- and rural-based anthropogenic activities.

As per the United States Environmental Protection Agency (US EPA), the volatile organic compounds are organic carbon compounds that cause a photochemical reaction in the atmosphere, low solubility in water, and readily vaporize into the air at room temperature. Industrial solvents like benzene, butane, toluene, esters, propane, pentane, methane, hexane, chlorohydrocarbon, trichlorofluoromethane, ketone, chloroform, acetate, etc., other industrial agents like lubricants, paints, petroleum fluids, dry cleaning chemicals, inks, varnishes, cosmetics, etc., classified as volatile organic compounds [3]. Chemical and petroleum industries are found to be the key sources in the expulsion of volatile organic compounds. Such volatile organic compounds pollute air followed by water and soil. Mainly ground-level ozone is generated by the interaction of volatile organic compounds with nitrogen oxides in the atmospheric layer. Further, smog was formed by the reaction of this ground-level ozone and volatile organic compounds [4]. Thus, produced ground-level ozone and smog had produced drastic changes in the climate, and environment, affecting the health of creatures. Heavy metals are defined as high-density substances ranging from 3.5 to 7 g/cm^3^ which even at low concentrations are toxic to living beings and damage aquatic ecosystems [5]. The main heavy metal sources are humans’ anthropogenic activities such as mining, discharges from metal-based industries, and domestic usages [5,6]. Notable heavy metals are arsenic (As), cadmium (Cd), chromium (Cr), cobalt (Co), copper (Cu), iron (Fe), lead (Pb), mercury (Hg), manganese (Mn), nickel (Ni), selenium (Se), silver (Ag), thallium (Tl), zinc (Zn), etc. [7]. Amidst arsenic, cadmium, chromium, lead, mercury, and silver can produce toxicity even at very low concentrations [5,8]. Heavy metals’ environmental persistent and non-biodegradable nature allows them to disturb the food chain. Heavy metals enter into the bio-system through the consumption of water and food loaded with contaminants [9]. While considering the bio-interaction mechanism of these heavy metals, the evolution of reactive oxygen species with free radicals produces oxidative stress in the cell components. Thus, the destruction of cells was observed through the damage of proteins, lipids, and nucleic acid by free radicals [6,8,10]. Even a very low concentration of heavy metal ions with a density of >6 g/cc have the potential to produce carcinogenic products in the living system, leading to cancer and consecutive death [9]. Figure 1 represents the various sources from urban and rural areas polluting the water sources through several anthropogenic activities.

## 2. Biofiltration techniques vs conventional techniques in the removal of VOCs and heavy metals

A major percentage of volatile organic compounds and heavy metals were introduced into water sources or the environment by industrial discharges. It is responsible for those industries to follow innovative systematic methods to remove volatile organic compounds and heavy metals from wastewater before releasing them into the environment or water sources [11]. Several nonbiological methods exist for the removal of different VOCs and heavy metals from polluted water. Conventional (nonbiological) wastewater treatment processes are physical, chemical, and biological process that include coagulation/flocculation, water screening, sedimentation, filtration, disinfection, electrolysis, activated sludge, etc. [12]. Techniques such as absorption, absorption in scrubbers, adsorption, advanced oxidation process (photocatalytic based), stripping, volatilization, condensation, ozonation, filtration, membrane separation, and incineration were employed in the case of removal of VOCs [11,12] (Table 1). Whereas in the removal of heavy metals, techniques utilized are adsorption, air stripping, chemical coagulation, chemical precipitation, electrochemical methods, ion exchange, membrane separation, and solvent extraction (Table 2). These conventional methods show off its limitation such as expensive, toxic secondary pollutant, high-quantity chemicals, need of skilled professionals, uninterrupted power and air supply, not suitable for dispense dyes, expensive regeneration process, not applicable for low concentration of pollutant, fouling, and release of ozone [12–15].

**Table 1.** Removal of volatile organic compounds with conventional methods

Nonbiological VOC removal method	Working mechanism	Pollutant	Catalyst	Inlet of VOC	Retention time	Removal efficiency of VOC	Advantages	Disadvantages	Reference
Absorption (wet scrubbing)	Scrubber absorbs the VOCs. This is applicable for water-soluble VOCs	Butanol	-	5 ppmv	20 ms (residence time)	90%	Regeneration of scrubbing liquid through an advanced oxidation process.	Pressure drop in packing structure. A secondary pollutant is a problem.	[23]
Incineration (Metal catalyst)	Laboratory scale tubular reactor used in the decomposition of pollutants in the presence of metal catalysts	1,2-Dichloroethane	Pt; Fe_2_O_3_	[C_2_H_4_Cl_2_]_0_ is the inletconcentration (mol/ L)	1.0 s	66–99.8%; 53–99% at 550°C	Control over retention time with operating temperature.	Disposal of waste is the problem. High energy consumption.	[24]
Ozonation	COF mineralization through hydroxyl radicals from the catalyst	Cooking oil fumes (COF)	Fe(OH)_3_	THC concentration of 211 ppm	0.05 s	95%	The oxidizing capability of ozone.	Extensive evaluation of catalyst performance for different VOCs.	[25]
Adsorption and ozonation	Adsorption of VOCs followed by oxidation	Methyl ethyl ketone	Alumina silicate	1.35 g m^3^	-	93%	Strong thermal and chemical stability.	Adsorbed byproducts decrease the adsorption capacity.	[26]
Adsorption andcondensation	Open-circuit and closed-loop flow in regeneration mode	VOCs	-	4099 ppmv	-	98.50%	Ozone and secondary organic aerosol production after VOCs reduction.	Checking theapplicability for urban areas.	[27]
Oxidization and volatilization	Anodic electrochemical oxidation of pollutants	Chloroform, benzene, trichloroethylene,and toluene	Pt/Ti, IrO_2_/TiIrO_2_/Ti, IrO_2_/TiIrO_2_/Ti, and IrO_2_− Ru−Pd/TiIrO_2_− Ru−Pd/Ti anodes are employed	150 mg/L	-	98%	Electrochemical properties of the anode in the removal of VOCs.	Selection of suitable anodes forparticular VOC. Disposal of catalyst is a problem.	[28]
Membrane separation	The capture of VOCs by a dense porous fibrous membrane	Aniline, benzene, and toluene	Poly(1-trimethylsilyl-1-propyne)	2 mL of VOC solvent	-	871 mg/g anilineadsorbed	Higher adsorption capacity.	Membranes are expensive. Aging of polymers.	[29]

**Table 2.** Removal of heavy metals with conventional methods

Conventional method	Adsorbent	Heavy metal	Observation	Efficiency	Advantages	Disadvantages	Reference
Adsorption	Graphene oxide-based microbots	Lead(II)	Cleaned water from 1000 ppb down to below 50 ppb in 60 min	95%	A wide range of heavy metals are removed. More removal efficiency. High specific surface area.	Expensive. Sludge production. Regeneration is not possible. Adsorbent decides the metal removal efficiency.	[30]
	Oxidized activated carbon	Copper(II)	Adsorption capacity increased with a pH range of 3.0–6.0	91.30%			[31]
	SiO_2_-Carbon nanotube	Mercury(II)	Endothermic process, mercury removal increased with increase in temperature	98%			[32]
	Polypyrrole-based activated carbon	Lead(II)	Highest adsorption at pH 5.5, followed chemisorption pathway.	81.80%			[33]
	Geopolymer from dolochar ash	Cobalt(II), nickel(II), cadmium(II), and lead(II)	The process was spontaneous and endothermic. Maximum removal at pH, temperature, and initial metal ion concentration were 7.8, 343 K, and 10 ppm.	98–99%			[34]
Air stripping		Nickel ammoniacomplexion	Optimal parameters pH 11, the temperature of 60°C, and an airflow rate of 0.12 m^3^/h	Nickel and ammonia were less than 0.2 mg/L and 2 mg/L	Low cost. Reliable technique.	Not suitable for a wide range of pollutants. Bulk pollutants could not be removed.	[35]
		Mercury	Air stripping with chemical reduction treats a large volume of water.	94% Decrease in mercury level during the injection.			[36]
Coagulation	Ferric chloride and alum	Arsenic	Not effectively remove As from the municipal wastewater to <2.00 μg/L	Reduced total recoverable arsenic from 2.84 and 8.61 μg/L	Dewatering, microbial inactivation, and sludge settling properties.	More sludge is produced. Requirement of chemicals.	[37]
	Humic-like component of terrestrial origin	Copper(II)	Enhanced removal efficiency by intermolecular bridging between the pollutant and humic component of molecular range 100 kDa0.45 μm.				[38]
	Iron electrode	Chromium(IV)	Sinusoidal alternating current reduces energy consumption and enhances removal efficiency.	99.73% and the residual Cr(VI) in the effluent was <0.1 mgdm^−3^			[39]
Chemical precipitation	Cu-EDTAdecomplexation	Copper	Cu ions were precipitated as Cu_2_(OH)_2_CO_3_, CuCO_3_, Cu(OH)_2_, and CuO.	68.30%	Low investment. Facile process.	More sludge is produced containing metals. High sludge and maintenance cost.	[40]
	Magnesium hydroxy carbonate	Oxovanadium(IV), chromium(III), and iron(III)	Removal efficiencies of heavy metals were increased with the dose of magnesium hydroxy carbonate (.30 g for 50 mL)	99.90%			[41]
Electrochemical	Graphene oxide electrode	Copper, cadmium, and lead	The high density of surface functional groups to assist the electrodeposition by the graphene oxide electrode	>99.9%	Pure metals can be recovered. No chemicals requirement. Rapid technique.	High capital and running costs. Generation of by-products.	[42]
		Zinc (Zn), nickel (Ni), and copper (Cu)	Electrochemical better than nanofiltration	99.81%, 99.99%, and 99.98%			[43]
Ion exchange	Li_1.9_MoS_2_	Mercury(II), lead(II), cadmium(II), and zinc(II)	Lithium-intercalated layered metal chalcogenides experience exfoliation when treated with water	580 mg of mercury/g	A wide range of heavy metals are removed. Appreciable regeneration and pH tolerance.	High capital and running costs. Only selective metals are removed.	[44]
	Carboxylic weak acids	Copper(II), iron(II), lead(II), and zinc(II)	The complexing nature of carboxylic weak acids stabilize metal ions in solutions generating broader functional pH regions for metal extraction.	Extraction >85%-99%			[45]
Membrane	Ceramic supported graphene oxide (GO)/Attapulgite (ATP)	Copper(II), nickel(II), lead(II), and cadmium(II)	The use of aluminum oxide substrate increased stability and extended usage of membrane	Rejection efficiency 99–100%	High efficiency toward metal selected. Less chemical consumption. Simple design that occupies less space.	Expensive. Fouling of membrane. Flow rates are less. Sludge production.	[46]
	Layered cellulose-based nanocomposite membrane	Silver, copper(II), iron(II), and iron(III)	The high affinity of the membrane toward metal ions.	86–100%			[47]

Currently, the implementation of safe green technologies was considered to remove various contaminants from water. Some of the aforementione drawbacks of the conventional water treatment methods can be rectified by the biological water treatment methods. Bio-based techniques are activated sludge, aerobic and anaerobic treatment, biological filters, trickling filters, bioreactors, biosorption, bioscrubber, biofilms, phytoaccumulation, phytoremediation, phytostabilization, phytovolatilization, microbial fuel cells, rhizodegradation, rotating biological contactors, and vermifiltration were found to be very effective in the treatment of contaminated water [13,15,16]. One of the green strategies – the bioelectrochemical process – was carried out mainly to yield energy while treating the wastewater. Nowadays, researchers are engaged in performing wastewater treatment by employing microbes through the bioelectrochemical process along with the credit of receiving energy [17,18]. Amidst, the given methodologies, biofilters are found to be suitable, sustainable technology, and easy to operate in the removal of various contaminants present in the aquatic environment. Biofilters as an important emerging technique utilize biological living things as catalysts such as algae, bacteria, plants, protozoa, viruses, yeast, and mixed microbes [15]. These biological filters are flexible such that required designs were constructed depending on space and capital. An environment-friendly biofilter process was chosen due to its merits over conventional water treatment techniques. Biofiltration techniques are cost-effective, safe, user-friendly, no evolution of secondary pollutant, less chemical usage, high flow rate, absence of external thermal power, applicable for various toxic pollutants, works at room temperature, eco-friendly, and a significant percentage of efficiency even for low concentration of contaminants [11,15,19]. A notable advantage of biofiltration techniques was that the contaminants were converted into biodegradable wastes without the evolution of secondary pollutants within a given time frame [19].

A sustainable future is possible only when the early natural biodiversity cycle got restored [19]. The anthropogenic activities against nature should be kept under control to avoid all types of pollution to regain globe of blue and green. Recent review articles are available on biofilters for the removal of volatile organic compounds and heavy metals from polluted air and wastewater [15,16,20–22,49–52]. The majority of the works concentrate on treating the gaseous pollutants present in the air by employing biological technologies [15,16,20–22,48,51]. Removal of pollutants from the air to maintain a healthy indoor environment by utilizing botanical filters was given [15,21,48]. Works are reported on de-odoring the gas streams during sewage treatment by microbial growth [16,20,22]. Precise work was drawn on biotechnologies for the extraction of organic solvents from wastewater released from metal refineries [49]. Another work had generalized the biodegradation of volatile organic compounds by biofiltration technologies to address the peaking down of the air quality [51]. Recent work is available on biological-based technologies in the treatment of wastewater [50]. In this work, biotrickling and bioscrubber are employed in the removal of VOCs. Whereas the botanical and biosorption methods find their potential in the capture of heavy metal [50]. This review article aroused to express in a wide manner the biological-based filtration techniques in the treatment of water, polluted by volatile organic compounds and heavy metals. Here, the review is concerned to gather the start-up with pieces of information on biofiltration technologies. After describing the global environmental issues followed by worthy of biological methods over conventional water treatment methods. Individual sections are included on the history, working mechanism, and influencing parameters for the successful operation of biofilters, which will be utilized by the student community, young researchers, engineers, and industrialists. Whereas the core sections differ from the existing reports by offering very recent trends and notable works on biofiltration methods in the treatment of wastewater. In this context, to the best of our knowledge, this is a review article that covers important amyloid-based membranes for the universal water treatment process including real industrial wastewater treatment. Also, the other related works are not missed out and are presented in a table format, which will act as a guide to researchers. Mainly, the core content was divided based on the type of biofilter setup or approach, which gives out the reasons, significance, and results (efficiency) of work taken into account for discussion in the removal of VOCs or capture of heavy metals. More prominence was shown to describe the working of every type of biofiltration technique along with its pros and cons for real-time employment. Finally, the review was concluded by addressing the key challenges to be rectified and future perspectives for biological-based filtration technologies in the removal of varied pollutants.

## 3. Biofiltration

### 3.1 History of biofiltration technique

Biofiltration is the biological-based technique employed to treat contaminated air and water [52,53]. The biofiltration process was carried out with the biological filters also known as biofilters. Biofilters consist of filter media where the microorganisms attach themselves and colonization takes place. These microorganisms are responsible for the oxidation and removal of pollutants present in air and water. As they tend to capture and degrade the organic and inorganic pollutants present in air and water [54]. In England in 1893, a pioneering bio trickling filter was used in the treatment of sewage wastewater [20]. In 1923, H. Bach German Scientist has reported on the use of living organisms to degrade the poisonous gas hydrogen sulfide present in wastewater [21]. In 1953, the first biofilter was implemented using microbiological growths in soil beds to control sewage odor in Long Beach, California, USA. The patent was issued for the method to Richard Pomeroy in the year 1957 [22]. After the 1950s, regular usage of biofiltration technology was started. A major focus was given to the treatment of toxic volatile compounds from the industrial sector by Europe and the US. For this, biofiltration setup was designed with appropriate filter beds and microorganisms [21,55]. In Europe and Japan, more than 500 biofilters had shown commercial success with good operating efficiency [56, 57]. A plethora of research works is reported on the application of biofiltration techniques in the decontamination of polluted air and water/wastewater. The merit of biofiltration in controlling volatile organic compounds with ~90% efficiency than the other existing pollution control technologies was reported [21,54,58].

### 3.2 Biofiltration mechanism in the removal of contaminants

**Figure 2.**
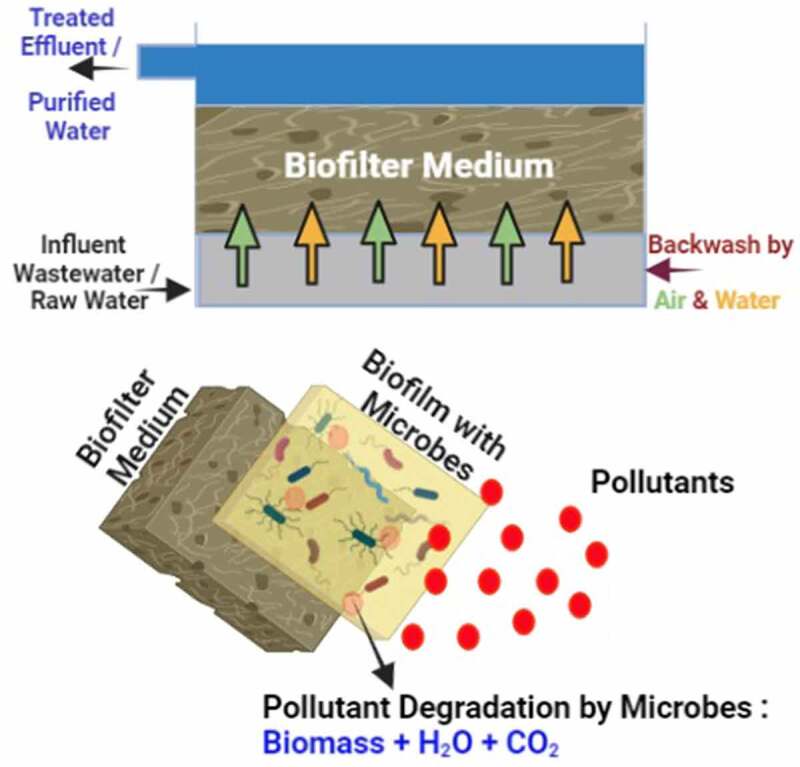
Biofilter typical setup and working mechanism in the degradation of organic and inorganic pollutants present in air and wastewater.

The biofiltration process consists of several steps in the removal of the contaminants from air or water. Initially, the contaminated air or water was given as input into the biofilter setup in which the contaminants are absorbed on the biofilm or cellular membrane of the biofilter bed. The transportation of the contaminants to bed media was done in the aqueous phase [52,59]. Contaminants as a source of carbon served as food to the microorganism. In turn through good metabolism, the microorganisms grow well forming colonies and resulting in the degradation of contaminants [52]. Finally, treated water or purified air were expelled out with the end products water, biomass, and carbon dioxide (Figure 2).

In simple, the biofiltration process can be represented as [20,60],

Pollutants + Biological creatures + Oxygen → Biomass + Water + Carbon dioxide (Eq. 1)

Two major steps or mechanisms involved in biofiltration are sorption and biodegradation [52,59]. The contaminant-filled air or water fed into the biofilter bed. Here, the contaminants phase transfer into an aqueous or solid phase in the biofilter media. Transformation of phase and continued degradation of contamination by microorganisms occur by below-given mechanisms [59].

- Adsorption of a contaminant into organic media (biofilter bed) followed by biodegradation by microorganisms.
- Direct adsorption of contaminant by biofilm and biodegradation.
- Dissolution of contaminants in aqueous phase and biodegradation.

The contaminants are removed from the biofilters after biodegradation [52].

The performance of biofilters depends upon the microorganism [61]. They are responsible for the phase transformation and degradation of contamination present in input polluted air or water. So, it is necessary to maintain the microbes to get efficient removal of contaminants. Recent research optimizes and develops genetically engineered microbes for safeguarding the environmental sources from toxic chemicals and metals [62,63]. The following section gives in detail the role of these biological creatures in the biofiltration techniques. So, the microbial community is restrained or immobilized in the biofilter bed. Immobilization of the microbes could be done in two ways. By natural attachment or artificial immobilization of microbes to the biofilter bed materials [61]. The purpose of microbial immobilization to achieve the high production rate of desired microbial product through improved survival rate of microbes by enhanced metabolism and increased cell loading in the filter bed material [64,65].

#### 3.2.1 Self-attachment of microorganisms

In the natural attachment, the microbes self-attach to the biofilter bed material. A higher concentration of microorganisms has to be maintained in the system as the naturally attached microbes’ concentration was higher than the suspended microorganism [66]. Better metabolism and biodegradation take place in the microbial biofilm when compared to that of the suspended microorganism system [67]. The glycocalyx is the layer of the cell membrane of microbes composed of polysaccharides. This mainly contributes to the structure of microbes and their attachment to the surface [66]. Recent research work shows that rhamnolipid released by microbes shows significant results in the formation of biofilm through microbial attachment in wastewater treatment [68]. A combination of forces acts in the self-attachment of microbes such as covalent bond formation, electrostatic interaction, and hydrophobic interaction. Further, the partial covalent bond between the hydroxyl group from the surface and microorganisms also participates [66,69]. These forces got varied depending on several factors like chosen microorganisms, the surface of filter bed material, environmental conditions, and fluid properties [66,69]. At the beginning of the adsorption process, the force due to electrostatic interaction was found to be higher than the other forces [67]. It was greatly accepted that filter bed material or pack made of organic substances greatly supports microbial development by providing nutrients [69]. In the case of inorganic materials – ceramic or glass – metal hydroxides are formed on their surface. These metal hydroxides are responsible for the arose of the partial covalent bonds [66,69].

#### 3.2.2 Artificial immobilization of microorganisms

Artificial immobilization of microorganisms takes place in five ways [66]. They are covalent bonding, covalent cross-linking, entrapment, membrane separation, and microencapsulation methods.

##### 3.2.2.1 Covalent bonding

In this method, microbial cells are directly attached to the water-insoluble carriers through a covalent bond. Inorganic materials, synthetic polymers, water-insoluble polysaccharides, and proteins are used as carriers [67]. Microbial cells consist of different reactive groups, which readily bind with ligands present in the biofilter bed material through a covalent bond [69]. Some of the reactive groups present in the microbial cells can create toxic effects. Hence, leakage of cell division occurs leading to the low percentage of attachment of microbes into biofilter media [66].

##### 3.2.2.2 Covalent cross-linking

The covalent cross-linking method is the extension of the covalent binding method in which the covalent bonds were formed between the microorganisms resulting in the formation of three-dimensional microbial structures. As discussed in the covalent bonding technique, the immobilization of microbe was disturbed by the toxicity from reactive groups [69].

##### 3.2.2.3 Entrapment

Microbial cells are entrapped into the three-dimensional polymer matrices. The matrices are made of polyester, polyurethane, polystyrene, cellulose, agar, resin, etc. The pores of polymer matrices are appeared to be smaller than microbial cells. Such that the microbial cells are trapped in it [67]. Entrapment immobilization of cells has certain merits such as plasmid stability, high metabolism of the entrapped cell, mild experimental condition, low cost, physically separated and immobilization of different microbes, restriction to toxic compounds, biodegradable and eco-friendly. The main demerits include the high diffusion restriction for some polymers, metabolic alterations, and less oxygen consumption in entrapped microbial cells, which leads to damage of those cells [66,70].

##### 3.2.2.4 Membrane separation

The membrane separation method was used to separate the microorganisms from a large liquid medium by utilizing membranes. Ultrafiltration membranes of porous nature of size 0.002–0.1 µm are used for the process. Also, non-porous membranes and special membranes could find their potential in the treatment of water [71]. The major drawback to be considered was fouling of membrane after treatment [67]. Physical and chemical conventional methods for cleaning the membrane might cause damage to immobilized microbial cells in the biofiltration system. A variety of composite membranes and technologies are reported on antifouling characteristics that were mainly employed in wastewater treatment and water purification [72,73].

##### 3.2.2.5 Microencapsulation

As the name suggests the microorganisms are wrapped in a droplet-shaped thin membrane. Within their capsule, the microorganism can move freely. Microencapsulation offers protection to microbes against environmental and mechanical stresses. Therefore, the microbes possess better metabolism and increased growth rate [64]. Looking into the diameter of the microcapsules ranges from 10 to 100 µm [67]. Due to this micro range of the capsule, the diffusion of substrates into the capsule and release of microbes’ metabolites out of the capsule is very easy [64,65]. Materials used for encapsulation include epoxy resins, cellulose nitrate, nylon, etc. [66,67]. The microencapsulation technique addresses the disadvantages faced in other immobilization techniques that count to low cell loading, decreased metabolism, cell leakage, contamination, and weak mechanical stability [64].

### 3.3 Parameters influencing biofiltration process

Several parameters that come under physical, chemical, and biological types determine the efficiency of the biofiltration process.

#### 3.3.1 Biological organisms

In the biofiltration process, the key ingredient is a biological creature that acts as the catalyst to initiate the process. Microorganisms like bacteria, protozoa, invertebrates, and fungi are used to form biofilm. Mostly, bacteria and fungi are considered for microbial communities in the filter beds. These heterotrophic microbial cells are immobilized to carry out the complete degradation of pollutants. Initially, the cells attach them to the surface of the biofilter bed in the reactor. Then, colonization of microbial cells takes place on the surface to form an active layer to capture the pollutant. This was done by the secretion of polysaccharides an extracellular component and arousal of covalent bonds with several surface interactions [67]. Another way is to attach the microbial cells artificially to the biofilter bed. Artificial immobilization of cells was carried out with micro capsulation, membrane, cross-linking, carrier bonding, and entrapment [67]. These microorganisms formed in the bioreactor bed are responsible for the odor control, degradation of organic and inorganic pollutants from the influents. One of the volatile organic compound toluene was eliminated by fungal-based biofiltration, which occurred to be more effective than the bacterial operation [74,75]. This was due to the resistant nature of fungi in a dry and acidic environment. Upon nutrient supply elimination capacity of toluene by fungi biofiltration was recorded to be 90 and 95 g/m^3^h for coconut fiber and compost biofilter [74]. Saprophytic fungus- *Phanerochaete chrysosporium* had shown removal efficiency in the range of 50–92% with an elimination capacity of 1913.7 mg/m^3^h [75]. In the other work, genera Enterobacter as a degrading species had produced the degradation rate of benzene from the waste gas stream of 21.46 g/m^3^h with a removal efficiency of about ~90% [76]. These microbial concentrations were found to be ~10-15% in the biofilter [77,78]. Recently, Actinobacteria (aerobic microorganisms) have shown their efficacy in the complete removal (100%) of odor and degradation of butyric acid [79]. The total bacterial count of 10^4^–10^10^ CFU/g is present in the biofilter compost bed [77,79,80,81]. Thus, the selection and concentration of microbes in the biofilter play a major role in the degradation of pollution.

#### 3.3.2 Biofilter bed

Next to microorganisms (biological creatures) biofilter bed or packing material is the heart of the biofiltration unit. Microorganisms are immobilized on the biofilter bed, which acts as media to grow as a biofilm [15]. Both organic and inorganic materials are used as the packing material of biofilter beds. All-time available materials such as soil, compost, wood chips, cocopeat, perlite, ceramics, polyurethane foam, etc. were employed to construct packing media at low cost [82]. These packing materials are expected to possess the following characteristics [83]:

1. A high porosity and specific surface area (300–1000 m^2^ m^−3^) to support the homogeneous distribution of influent.
2. Rich in intrinsic nutrients to enhance the growth of microorganisms to form biofilm.
3. Intense presence of different microorganisms.
4. Better water retention capacity (40–60%) to enhance the metabolism of microorganisms.
5. Mechanical and thermal stability to avoid packing down of filter bed.

Soil is a natural packing material. Although it possesses less intrinsic nutrients, it holds various microorganisms with high specific areas. Whereas the peat holds a high specific area with less amount of intrinsic nutrients and microorganisms [15]. Composts are considered for their characteristics such as the presence of intrinsic nutrients, water retention capacity, dense presence of various microorganisms, and good air permeability [15]. Due to the high water retention behavior, the compost disintegrates and results in a pressure drop. Good stability followed by biodegradation was achieved in the wood chips [83]. On the other hand, low specific surface area, low nutrient, pressure drop, and low pH buffering capacity were observed. However, wood chips are considered for their pollutant efficiency by varying the loading concentration [83]. Ceramic materials like biofoam and perlite have good thermal stability and offer low resistance to gas flow [82]. Four different packing materials such as cattle bone porcelite, horticultural porcelite, open-pore polyurethane foam, and perlite were compared for their performance in the removal of toluene [82]. Amidst the cattle, bone porcelite had exhibited maximum removal efficiency of ~75-80 g m^−3^ h^−1^ for a gas retention time of 13.5 s at a critical load of 29 g m^−3^ h^−1^. The biofilter bed made of cattle bone porcelite elongates its consistent performance for 5 months without any pressure drop. Other filter bed materials – pumice and coke – were observed for their high porosity nature causing trouble in cleaning and giving rise to uncontrolled growth of microorganisms [84]. A plethora of research works was reported on various biofilter beds for a stable structure and pollutant removal efficiency. Sugarcane bagasse as a filter bed packing material inoculated with Hyphomicrobium VS and Thiobacillus thioparus Tk-m were utilized in the removal of dimethyl sulfide. Hyphomicrobium VS had produced an efficiency rate of 97.6% at an inlet concentration of dimethyl sulfide of 12 ppmv [85]. Recent work was demonstrated with lignocellulosic residues such as rice husk, sugarcane bagasse, pruning waste, and chicken manure as filter bed materials. Mixtures of rice husk and sugarcane bagasse have shown the highest elimination of ammonia and hydrogen sulfide >95% [86]. Integration of activated carbon to filter bed material has shown increased biodegradation of pollutants [87,88].

#### 3.3.3 Supply of nutrients

The nutrient is another important parameter to decide on the efficiency of biofiltration. The microorganisms degrade the pollutants feed into the biofilter. These pollutants provide energy to the microbes by acting as a source of carbon. The essential micronutrients and macronutrients are achieved from the supporting pack materials utilized in the biofilter bed. Nitrogen and phosphorous are the major macronutrients along with potassium and sulfur, whereas micronutrient includes metals and vitamins [89]. These nutrients are introduced into the filter bed in the solid or liquid phase. Mostly mineral salts are dissolved in an aqueous solution and used as a nutrient solution in the biofilter bed. Frequently used mineral salts are, CaCl_2_, FeSO_4_, KH_2_PO_4_, KNO_3_, (NH_4_)_2_SO_4_, MgSO_4_, MnSO_4_, NH_4_Cl, NH_4_HCO_3_ and Na_2_MoO_4_ [11,15]. Many research reports are available that show the supply of nutrients supports the growth of microorganism [89,90]. In the presence and absence of nutrient supply, the removal of butanal from polluted air was carried out with biofilters. The results show that biofilter with nutrients had yielded 97% of elimination efficiency, whereas 86% was produced by biofilter without nutrients [89]. Further, the suitable packing material chosen for the biofiltration bed is important in enhancing microbial activity. The surplus nutrient was available from sludge-based organic compost material providing macronutrients to the microbes. On the other hand, synthetic or inorganic materials consist of less or no nutrient content [91]. Several studies were performed in choosing the packing material for the significant removal of contaminants. These studies proved the betterment usage of organic packing material when compared to inorganic materials [92,93].

#### 3.3.4 Power of hydrogen (pH)

Another important factor is pH which equally has importance over biofiltration performance. Nutrients and their role in the effective degradation of pollutants by microbes are possible under the optimum value of pH [94,95]. The heterotrophic microbes that thrive in the biofilters are neutrophilic organisms, i.e., the living environment possesses neutral pH of 7. Many studies stand as proof of the pH effect in the removal efficiency of contaminants. The study was reported on the effect of oxidation of methane by hydrogen sulfide at different pHs. It was recorded that the flux rate for oxidation of methane was 53 g/m^2^/day at acidic pH (4.5), whereas 146 g/m^2^/day at neutral pH (7.0) [95]. Acidification takes place by the secretion of sulfuric acid while biofiltering the reduced sulfur compound and dimethyl sulfide. This acidification makes the variation in pH and decreases the performance of biofilter. Hence, methanol was added to avoid sulphuric acid production and maintain the pH in the reactor [96]. Recently, bioaerosol emission was studied at low and neutral pH of biofilters in treating the odors that occur from landfills. At a high inlet flow rate, the removal efficiency was more with heterotrophic bacteria and fungi for low and neutral pH, respectively, [97]. Followingly, the same research group has proposed the biofilter treatment of contaminated gas containing acetic acid, ammonia, hydrogen sulfide, and toluene with low and neutral pH [98]. Acetic acid and ammonia were removed at an efficiency rate of 99.92% and 99.90% under neutral pH by microbial degradation. For hydrogen sulfide and toluene, the higher removal efficiency was observed as 99.24% and 99.90% with low pH. The high-pressure drop occurs in low pH conditions due to the presence of fungi [98]. Removal of a combination of aromatic benzene, toluene, ethylbenzene, and *o*-xylene (BTEX) from contaminated gas stream gains importance. A plethora of research works were performed on the biofiltration of BTEX. The experiment was performed in the degradation of benzene and *o*-xylene at neutral and acidic pH using heterotrophic microbes (bacteria and fungi) for biodegradation in the presence of surfactants (Brij 35, Saponin and Tween 20) [47]. Pseudo-first-order kinetics was applied in the biodegradation of benzene and *o*-xylene. Fitted data show that neutral pH (7) was more effective in the removal of benzene and *o*-xylene than acidic pH (4). The unnamed culture of microbes was observed at acidic pH4 with an increase in benzene (11%) and *o*-xylene (22%) than at neutral pH [99]. These studies denote the influence of pH on the degradation of contaminants by microbes and the maintenance of optimum pH without any disturbance.

#### 3.3.5 Operating temperature

The operating temperature of the biofilter determines the number of active microbes followed by the degradation of pollutants. Effect of temperature in the removal of natural organic matter by drinking water biofilter was reported [100]. The operating temperatures of 5°C, 20°C, and 35°C were applied to the water surface. A decrease in the removal of organic matter was observed at a low temperature of 5°C. This effect was due to the change in the microbial structure affecting the metabolism rate of the substrate. Biofilters operating at 20°C and 35°C had produced parallel removal efficiency as produced by the disinfectants [100]. Removal of volatile organic compounds from indoor air by biofilter at warm and cool temperatures was reported [101]. Biofilters operating at cool temperatures have shown better outcomes like reducing the activity of microbes (like Legionella) and avoiding the negative impact produced by water vapor. When biofilter was held up at warm temperature the building get damaged through the internal air quality [101]. Biofilter fixed with *Pseudomonas putida* was used in treating waste air containing ethanol. As per previous reports, *Pseudomonas putida* had shown the optimum incubation temperature of ~26°C. The observation of microbial growth activity was demonstrated with the incubation temperature range 20–40°C. Now, it was interesting to note that the optimum incubation temperature of biofilter holding *Pseudomonas putida* lies at 30°C. As the biodegradation of ethanol was recorded to be 140 g/m^3^/h at 30°C, which occurred to be higher than other observed temperatures (25°C, 35°C, and 40°C) [102]. The degradation of endocrine disruptors and pharmaceuticals (*N,N*-diethyl-meta-toluamide, ibuprofen, and naproxen) for the wide range of annual temperature with biofiltration was reported [103]. From the pseudo first-order data analysis, it was clearly shown that at low (1°C) and intermediate temperature (12–15°C) the rate constant values are similar. But for high temperature (18–21°C) the lower bound estimation was observed which represents the concentration of effluent to be lower than the standard limit. Recently, work was proposed on the removal of nitrogen from municipal water for a range of temperatures by employing sulfur-limestone autotrophic denitrification biofilter (SLADB) [104]. It was observed that for the low range of temperature 6.4–9.8°C the total nitrogen (TN) and nitrate-nitrogen (NO_3_-N) were removed with a good efficiency rate of 81% and 85%, respectively. This was due to the pattern formed by the microbial community in a bioreactor with the applied temperature. Heterotrophic bacteria – *Anaerolineae* – which supports the heterotrophic denitrification were increased, whereas *Ferritrophicum*, *Sulfurimonas*, and *Thiobacillus* (supporting sulfur autotrophic denitrification) got decreased [104]. Very recent styrene (concentration of 20–150 ppm) removal from wastewater was demonstrated with ethanol as co-solvent at temperatures 15°C, 25°C, and 35°C. For the temperature 35°C, the styrene was removed at the rate of 93% and methane yield was recorded to be 4.14 [105]. Hence, it was comprehensible dependence of the biofiltration technique over temperature and the need for clear analyses to proceed with a new approach.

#### 3.3.6 Moisture contents

The base of biofilter performance depends on the activity of microorganisms. To obtain a significant biodegradation rate, the homogenous spread or growth of microbes as a biofilm on the biofilter bed is inevitable [106]. This could be achieved only by the presence of water content. The moisture provided by the water content helps the transfer of nutrients to microbes. An appropriate metabolic activity or degradation was carried out toward the contaminants from the influent [107]. Biofilter media whether its organic or inorganic nature determines the availability of moisture from water content. Organic and inorganic media are hydrophobic and hydrophilic, respectively [107]. Inorganic media retains the moisture content than organic media. So, the volumetric analysis was done to calculate the moisture content of the biofilter [78,107]. To avoid confusion over the dry and wet weight basis method. To obtain a good degradation rate, the optimal value of biofilter moisture content is in the range of −0.2 to −3 bars [107]. Research works are proposed on a hybrid of organic and inorganic material substrates to improve the contaminant removal [108,109]. These filtering materials possess different surface areas, optimal water content, and porosity. Ceramsite and lava rock from the inorganic groups along with fibrous material were utilized in the construction of the biofilter. This hybrid biofilter is used in the treatment of contaminated river water [108]. Organic material had shown ~44% denitrification than inorganic. Higher rates of ammonium (87–97%) and phosphorous (76–94%) were removed with inorganic materials [108]. Inlet gas temperature, pollutant oxidation (removal time) by metabolic activity, and pre-humidification of inlet gas/air are the considerable parameters to maintain the moisture of biofilter bed material [109].

#### 3.3.7 Pressure drop

Pressure drop of biofilter bed is another parameter in the degradation of pollutants. The relationship between pressure drop and biomass concentration was used in the prediction of the performance and stability of biofilter [110]. Polyurethane biomass filter has shown the pressure drop of 30–33 mm H_2_O/m for the biomass concentration 2.00–2.05 g-DCW g/PU [110]. Also, other factors such as flow rate, moisture content, and characteristics of biofilter media influence over pressure drop [111,112]. Biofilter performance in the removal of volatile organic compounds from waste gas was studied by ozone injection. Two biofilters with and without ozone injection were operated to record the efficiency of removal of toluene [113]. Biofilter injected with ozone possesses a lower pressure drop than the biofilter without injection. No change in the removal rate of toluene was noted during the biofiltration process. However, ozone exposure had avoided accumulation biomass and improved the biofilter removal rate by increasing microbial community for operation [113].

## 4. Biofiltration technique in the removal of volatile organic compounds (VOCs)

In this section, different types of biofiltration techniques engaged in the removal of several volatile organic compounds released from various industries such as dye industries, pharmaceutical industry, oil mills, petroleum refineries, etc., Before discarding the industrial wastewater, it has to be processed to remove the volatile organic compound which might be toxic and odor-causing substances. These volatile organic compounds cause pollution to air, water, and soil. Laboratory research experimental works were done with biofilter techniques for various chemicals such as toluene, benzene, ethylbenzene, xylene, styrene, *a*-pinene, etc. [76,99,101,113]. As discussed in the previous section, the performance of biofilters depends upon their parameter values during operation. Table 3 provides the various biological processes, which were employed in the removal of VOCs.

**Table 3.** Various biological-based processes employed in the removal of volatile organic compounds

VOCs	Methods	Sources	Elimination Capacity	References
BTEX	Biofiltration	*Paecilomyces variotii*	110 gC m^3^ h^1^	[131]
Toluene/styrene	Fixed-film bioscrubber	*Microbacterium esteraromaticum* SBS1–7	203 g·m^−3^·h^−1^	[132]
BTEX	Biodegradation	*Variovorax paradoxus*	71.3% of ethylbenzene, 61.1% of *m*-xylene and 54.8% of *p*-xylene	[133]
2-Ethyl-1-hexanol	Biotrickling filtration	Fungi and Bacillus Subtilis	95–98%	[134]
*n*-Hexane and dichloromethane	Biotrickling fil tration	*Mycobacterium* sp. and *Hyphomicrobium* sp.	12.68 g m^−3^ h^−1^ *n*-hexane and 30.28 g m^−3^ h^−1^ dichloromethane	[135]
*n*-Hexane	Biofiltration	Fungal biomass	3000 CFU/ml (optimum biomass)	[136]
Benzene	Biofiltration	*Aspergillus*	151.67 g m^−3^ h^−1^	[137]
Cyclo hexane and methyl acetate	Biotrickling filtration	*Ochrobactrum intermedium*	100%	[138]
Hydrogen sulfide (H_2_S), methanethiol, dimethyl sulfide, and dimethyl disulfide	Biotrickling filtration	*Acidithiobacillus*, *Metallibacterium*, and *Thionomas*	90.1%, 88.4%, 85.8%, and 61.8%	[139]
2,5-Dimethylpyrazine	Biofiltration	*Fusarium solani*	8.5 g m^−3^ h^−1^	[140]
Toluene	Biofiltration	*Scedosporium apiospermum*	258 g m^−3^ h^−1^	[141]
Phenol	Biofilter	Anaerobic microorganisms	>85%	[142]
Phenol	Biodegradation	*Acinetobacter* sp., *Pseudomonas* sp., *Nitrospira* sp., *Rubrivivax* sp.	~100%	[143]
Phenol	Biofilter	Microorganisms from municipal waste	~100%	[144]
Phenol	Biofilters	Chloroflexi and Planctomycetes	100 mg/L (effluent)	[145]
Toluene	Biotrickling filter	Cladophialophora	264.4 g m^−3^·h^−1^	[146]
2-Butoxyethanol	Biotrickling filter	*Pseudomonas vancouverensis*	72.8%	[147]
*n*-Hexane	Biotrickling filter	Toluene and 4-methyl-2-pentanone	10 g m^−3^ h^−1^	[148]
Benzene, toluene, xylene, and styrene	Biotrickling filter	*Burkholderia*, with little *Achromobacte*	90%	[149]
Toluene	Biotrickling filter	Pseudomonadaceae and Comamonadaceae	99.2%	[150]
Toluene	Biotrickling filter	Cell biochar beads seeded with *Pseudomonas* sp.	1134 g toluene/m^3^. day	[151]
Toluene	Biotrickling filter	Fungi Rhamnolipids	176.8 g m^−3^ h^−1^ ; 114 g m^−3^ h^−1^ (rhamnolipids)	[152]
Toluene	Biotrickling filter	*Fusarium oxysporum*	98.1 g m^−3^ h^−1^	[153]
BTEX	Biofilter	Microbial growth enhanced by polyurethane	61%	[154]
Ethylbenzene	Biofilter	Bacterias	-	[155]
Sulfur dioxide and *o-*xylene	Biofilter	*Pseudomonas* sp., *Paenibacillus* sp., and *Bacillus* sp.	96.09%	[156]

### 4.1 Biotrickling filter

**Figure 3.**
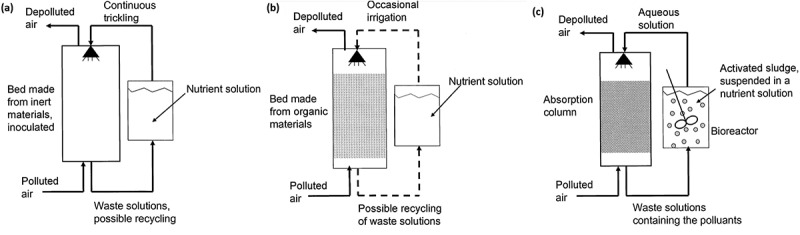
Typical schematic diagram representing (a) biotrickling filter, (b) biofilter, (c) bioscrubber working principle [15].

Biotrickling filtration setup consists of fixed microorganisms immobilized on the filter bed media that received continuous irrigation through an aqueous medium. The nutrient solution was given as input for the growth of microbes. Polluted influent goes through this medium. Degradation of these pollutants was carried out after their absorption on the biofilm [15] (Figure 3a). Biotrickling filters are employed as individual or hybrid methods with other existing techniques. This method was very efficient up to ~90% in the removal of the volatile organic compounds while compared to the other technologies like regenerative catalytic oxidation and adsorption methods [114]. The biotrickling technology was employed to remove the volatile organic compounds emitted from the chemical fiber wastewater treatment setup. Various microorganisms present in the biotrickling filter should be consistent and need an appropriate degradation time for the entire removal of contaminants [114]. The contaminant gas flow was made to flow from the bottom of biotrickling filters. The high population of microbes was found at low layer than the middle and upper layers of the biofilter. Empty bed residence time lies in the range of 32–59 s satisfying the national standards in contaminant concentration. Further, microorganism types and their distribution decide the outlet concentration of pollutants from the biofilters [114]. Biomass accumulation in the biotrickling filter affects the performance. This was overcome by full medium fluidization [115]. The removal of toluene by the hybrid trickle bed biofilter consisting of two mediums (pelletized and monolithic channelized mediums) was performed [115]. The pelletized medium had produced better activity than the monolithic channelized medium. The pelletized medium had shown efficiency till the accumulation of biomass. In this, biomass accumulation was avoided with backwashing for 320 min. No back pressure was observed between consecutive backwashing procedures. An efficiency rate of 99% was recorded for three runs of operation (toluene concentration 0.725 kg COD/m^3^/day). With empty bed residence time (EBRT) of 1-min heavy loading of toluene 2.27 kg, COD/m^3^/day was carried out [115]. On the other hand, the *n*-hexane removal rate was appreciable with a gas biotrickling filter. This was achieved with an increase in the inlet concentration and decreasing the empty bed residence time. The extracellular polymeric component in the biofilm increases the protein content (87.45–190.5 mg/g MLSS) which in turn helps for the effective removal of *n*-hexane. As the extracellular polymeric substance which covers the cell surface and decreases in biofilm’s negative charge density. This leads to biofilm formation by the aggregation of microorganisms. This activity well supported the biofilm growth and biomass accumulation in biofilter bed media. So, the stable performance of this biotrickling filter was obtained. This work had given out an elimination capacity of 45.36 g m^−3^ h^−1^ for an inlet concentration of 350 mg/m^3^ and gas empty bed retention time (EBRT) of 30 s [116].

By adding the specific strains of bacteria, the removal rate of VOCs could be improved in biotrickling filters. The bacterial strains are chosen to exhibit characteristics like high persistence, compatibility, and dominance in the degradation of contamination effectively. Toluene, dichloromethane, and *o*-xylene were removed with biotrickling filters inoculated with the two strains namely *Zoogloea resiniphila* HJ1 and *Methylobacterium rhodesianum* H13 with the removal rate of ~96.5% [117]. A maximum removal efficiency rate of 96.5% was observed after 17 days of interaction for the influent concentration of 450–600 mg/m^3^ and an empty bed retention time (EBRT) of 30–75 s [117]. Recently, a computational fluid dynamic model coupled with microscopic mass transfer-biodegradation kinetics model and macroscopic fluid model was proposed [118]. From which the modification and optimization of the existing biotrickling filter are possible. Hydrogen sulfide was removed with this computational fluid dynamic model. The removal efficiency was found to be increased with the increase in the size of biofilm (decrease of filter bed voids) and then decrease due to the clogging effect of biofilm. The microscopic mass transfer biodegradation kinetics depends upon the diffusion mass transfer and thickness of the biofilm. This microscopic mass transfer biodegradation kinetics is considered as the quasi-steady-state analysis. The proposed computational fluid dynamic experimental model goes well with parameters (concentration of pollution, pressure drop) of industrial biotrickling filters [118]. The presence of polyhedral spheres in biotrickling filters had shown simultaneous removal of hydrogen sulfide and ammonia with efficient compost deodorization. The theoretical evaluation was done to identify the microbial community engaged in the degradation of pollutants [119]. However, the microbial metabolism pathway had required a deep insight to expand the technique from lab or pilot scale to application scale. Likewise, odor-creating tannery emissions were controlled by the capture of hydrogen sulfide and ammonia with biofilters. The proteobacteria along with Acinetobacter and Firmicutes had shown enhanced removal efficiency of >99% for hydrogen sulfide and ammonia [120].

Also, biotrickling was utilized to remove carbon, methane, and other nutrients present in the municipal wastewater treatment plants [121,122]. Biotrickling filter coupled with chemically enhanced primary treatment (CEPT) had shown less operating cost and energy consumption. In municipal wastewater, the solid wastes and other organic loadings are removed with cationic polyelectrolyte and polyaluminium chloride (PAC) through coagulation-flocculation processes. Whereas it is interesting to note that the addition of a biotrickling filter contributes to the significant improvement of the removal of nutrients and carbon from municipal and industrial wastewater treatment. The result shows that total chemical oxygen demand of 89%, the biochemical oxygen demand of 94%, total suspended solids of 96%, PO_4_^3-^ -P of 78%, NH_4_^+^-N of 60%, and volatile suspended solids of 96% [121]. The efficiency of the enhanced result might be due to the microbes-based treatment of the biotrickling filter.

Wastewater resulting from anthropogenic activities was the greater source of methane emission globally. Methane presents in effluent gases from wastewater treatment plants are bio-oxidized by the biotrickling filter technology [122]. Methane is the substance that readily dissolves in water, i.e., hydrophilic nature. Easily taken as a nutrient by methanol degrading microbes in the filter. Upon increase in methane degrading microbes which in turn decrease in *a*-pinene degrading microbes. The work was demonstrated to remove the hydrophilic (methanol) and hydrophobic (*α*-pinene) volatile organic compounds under transient performance of biofilters [123]. In this, the mixture of wood chips and compost from mushroom waste acts as a biofilter media. The absorption process starts to remove the methanol followed by microbial degradation. However, the inlet concentration of methanol has affected the α-pinene degradation. Due to the hindrance produced by methanol toward the growth of microbial community responsible for *α*-pinene removal. Since methanol is hydrophilic degradation happens in a few hours. Whereas α-pinene is hydrophobic and removal can happen after 7–10 days. Although methanol concentration affects the *α*-pinene, degradation the concentration of *α*-pinene has not affected the methanol degradation. Significant results are possible by knowing the degradation activities of the microbial community for different substances and their impact on each other [123]. Similar work on mesophilic methanol and mesophilic *α*-pinene was carried out with a biotrickling filter for biodegradation at high temperatures. Methanol has a high degree of redundancy of functionality whereas *α*-pinene possesses unique property. The removal rates of methanol and *a*-pinene were 100 and 60 g m^−3^ h^−1^ at temperatures up to 70°C and 60°C, respectively, [124]. The results show that the biofiltration can be carried out at higher temperatures (>40°C) ie., applicable to hot gas streams with pollutants. DNA smear test which shows the fingerprint of microbes was used to analyze the microbial communities in the biofilter. Further, the study records the very important problem that arises on the channelling effect projected due to the overload of biomass and the addition of microbes [124].

The biotrickling filters can be utilized effectively when the following challenges are addressed properly:

- An accumulation of biomass in the filter bed
- Suitable selection of microbes
- Biofilm thickness
- Solubility of VOCs
- High operating costs for nutrient solution recycling
- Production of waste trickling liquid stream
- Complex operation

### 4.2 Biofiltration

A biofilter is made of a fixed filter bed in a bioreactor. In this biofilter bed, as described in the biotrickling biofilter, the microorganisms got immobilized. The influent with contaminants was sent and degradation of VOCs occurs by the metabolism of the microbial cells in the filter bed (Figure 3b). Two types of biofiltration setup are open-designed biofilters and closed-designed biofilters. Open-designed biofilters can experience climate change, whereas the closed one is kept inside the closed room. Another difference was in open-designed biofilters, the contaminated influent passes in ascending manner, whereas ascending or descending gas flow takes place in close-designed biofilters.

#### 4.2.1 Rotating drum biofilter

The rotating drum filter is considered for its merits like low manpower, removal of liquid from discharge, high volume, and variation in speed of drum [12]. It consists of a rotating hollow metal drum with a length of 1–20 feet, which rotates at the rate of one rotation per minute. The drum was covered by a filter cloth. Separate vacuum cells are present in the face of the drum and partially immersed in the contaminated slurry. When the drum rotates in solid or liquid suspension. In the separation of contaminant process, the slurry got sucked on the cloth and formed as a cake. Then, the dried cake can be removed using the drying process or washed with sprays [125]. Biotrickling, bioscrubbing, and other biofilter technologies have shown less oxygen mass transfer. This demerit was eradicated by rotating biological biofilter, which was evolved from the rotating biological contactor. In rotating drum biological biofilters, the high oxygen mass transfer and enhanced surface area are in contact with microorganisms. Though initial capital was high to construct a rotating drum filter, it can be maintained and operated at a low cost. Further, the rotating drum biofilter is suitable for the large-scale elimination of VOCs in industrial applications [126,127]. In a rotating drum filter, the key point is to add external nutrients such as phosphorous and nitrogen in addition to contaminants (VOCs) [127].

**Figure 4.**
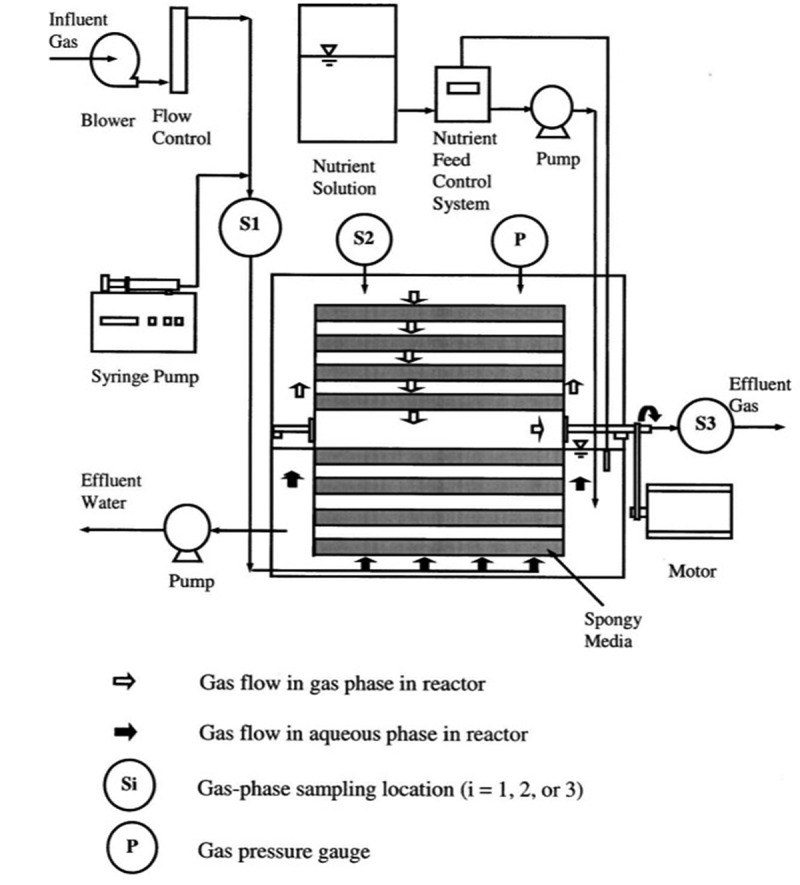
Schematic diagram representing hybrid rotating drum biofilter [126].

A hybrid bioreactor combining rotating drum biofilter (RDB) and an activated sludge process (ASP) was developed to remove toluene [126] (Figure 4). A single rotating drum biofilter without an activated sludge process could not yield biodegradation percentage as produced by hybrid (RDB-ASP). This was due to the reason that some amounts of volatile organic compounds were degraded by the activated sludge process. However, the removal efficiency of the rotating drum filter portion was higher than the activated sludge portion. In this work, upon increasing the organic loading rate from 1.58 to 6.32 kg, the biomass accumulation too increased at various medium depths. The organic loading rate depends upon the volume of the rotating drum. It was found to be high for a simple rotating drum process than the hybrid rotating drum biofilter. The biomass accumulation was decreased by removing the outermost biofilm layer of the rotating drum. This hybrid model had produced maximum toluene removal efficiency of 99.8% for 1.58 kg chemical oxygen demand/m^3^/day for EBRT of 38 s with 1 rpm rotating speed of drum [126]. Rotating biofilter was employed in the removal of the heavy concentrations of benzene, toluene, ethylbenzene, and xylene (BTEX) [127]. A smaller flow rate the rotating drum biofilter had produced improved removal efficiency with increased carbon dioxide concentration. Toluene was degraded with higher removal efficiency (86%) followed by ethylbenzene (83%), benzene (80%), and xylene (78%). In rotating drum biological filters, the removal efficiency of VOCs was found to be reduced with the reduction in empty bed contact time, increase in flow rate, and concentration of pollutants [127].

Limitations found in rotating drum filters are as follows:

- High initial investment to vacuum cells and the filter.
- Rotating drum filter not suitable for materials that form water-resistant cakes, i.e., cakes cannot be removed from filter cloth.
- Filtering hot liquids was hard if it starts to boil.
- Maximum atmospheric pressure difference was less than 1.

#### 4.2.2 Submerged aerated biofilter

**Figure 5.**
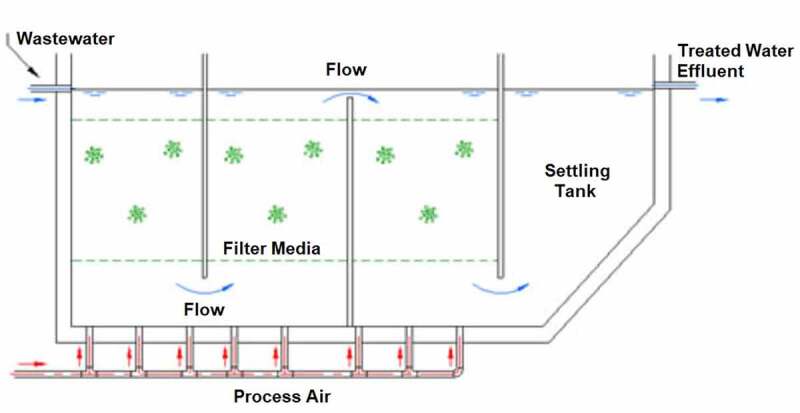
Typical submerged aerated biofilter setup for the treatment of wastewater [129].

**Figure 6.**
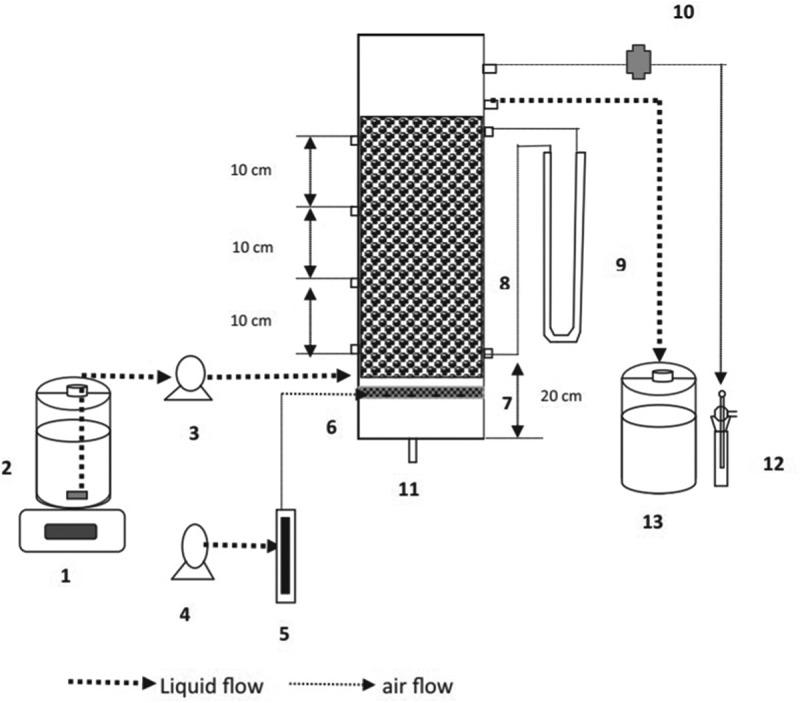
Schematic diagram representing the submerged aerated biofilter setup for the treatment of volatile organic compounds in pharmaceutical wastewater. (1) Magnetic stirrer, (2) Influent tank (3) Peristaltic pump, (4) Aquarium air pump, (5) Airflow meter, (6) Air inlet port, (7) Diffuser arrangement, (8) Packing media, (9) Manometer, (10) Connector for gas sampling, (11) Liquid drainage port, (12) Impinger, (13) Effluent collection tank [128].

The submerged aerated biofilters are easy to handle, ready uptake of nutrients by microbes, low sludge production, and odor-free system. Hence, submerged aerated biofilters are considered in the removal of pollutants. It is designed by arranging the series of cells through which the contaminated water flows and reaches the settling tank at the end. Contaminants that slough from the filter of each cell was removed in the settling tank. The setup was supplied with oxygen through the blower fixed at the bottom. So, the oxidation process got improved, mixing up the effluents efficiently and avoiding disturbances by excess solids from filters. The submerged aerated biofilter setup was employed in the biofiltration of pharmaceutical wastewater [128] (Figures 5 and Figures 6). With an increase in organic loading rate (OLR), the decrease in enzyme inactivation occurs, which leads to a reduction in removal efficiency. Volatile organic compound emission rate was monitored with hydraulic retention time, airflow rate, and organic loading. The volatile organic compound degradation efficiencies were 95% and 72% for organic loading rates of 17.45 ± 0.01  and 20.85 ± 0.03 kg/m^3^/day, respectively. Submerged aerated biofilter had produced maximum chemical oxygen demand (COD) of ~92% for the hydraulic retention time of 12 h and organic loading rate of 3.09 ± 0.05 kg/m^3^/day. Less efficiency was produced with overloading of biofilter at short hydraulic retention time. This was due to the inhibitory effect caused to the heterotrophic microbes (bacteria) in the bioreactor. For 2 h of hydraulic retention time the pollutant concentrations were 73 ± 0.48 mg/L (dichloromethane), 72.97 ± 0.89 mg/L (benzene), 72.33 ± 1.08 mg/L (toluene), 57.94 ± 1.56 mg/L (methanol) and 51.31 ± 1.59 mg/L (acetone).

When the contact time between the microbe and pollutant got decreased the degradation efficiencies also got decreased. The wide range of volatile chemical contaminants even at shock loading conditions might be improved with these submerged biofilters [128]. Sewage consisting mainly of urine requires an efficient treatment process. The bioreactor fixed with submerged aerator filter bed was employed for this typical domestic sewage wastewater treatment [130]. Phosphorous and nitrogen from wastewater were not removed >15%. As the ammonia nitrogen concentration in the wastewater affects the nitrifying bacterial growth by forming the ammonia. Further, pH (~9) got increased which was above the tolerance level of microorganisms. The study has to be analyzed in-depth to find out a solution for high ammonium concentration and high-level pH in turn causing microorganism detriment [130]. However, limitations like the requirement of additional units to remove phosphorous and constrained flexibility due to changing effluents.

#### 4.2.3 Other biofiltration works

Biofilters like anticlogging, botanical, regenerative, self-sustained, hybrids, etc. are developed in the removal of VOCs. Loading of contaminants in the biofilter plays an important role in the removal of contaminants. Water content in the biofilter determines the growth and metabolism of the microorganisms. In the biofiltration system, the packing materials remain in neutral pH of ~7 due to nitrification process [157]. Two biofilters with the same construction and design were taken for the study in the removal of VOCs from reformulated paint [158]. The gas stream chose was composed of acetone (450 ppm_v_), ethylbenzene (10 ppm_v_), methyl ethyl ketone (12 ppm_v_), *p*-xylene (10 ppm_v_), and toluene (29 ppm_v_). The role of intermittent contaminant loading and start-up strategies were observed in targeting contaminants. Biofilters made of polyurethane foam as a supporting medium were loaded with an enrichment culture obtained from municipal wastewater sludge and wood waste compost. The first biofilter was supplied with the contaminant loading 8 h/day (intermittent loading), whereas the other biofilter was loaded continuously with the contaminants. At EBRT time of 59 s with the start-up strategies for the contaminant loading rate of 80.3 g m^−3^ h^−1^ had given a higher removal rate of 99% for both biofilters. However, the first biofilter which experienced intermittent loading of contaminant had required a longer time to produce higher efficiency [158]. Hence, it was well understood that intermittent loading affects the performance of biofilter to remove the VOCs. Extra consideration was given in the case of complex mixture compounds present in polluted airstreams. Volatile organic compounds have different characteristics like aqueous solubility, biodegradation, and molecular steric hindrance. Multi-compound biodegradation was possible by employing the stratification technique of biodegradation in biofilters [159]. In this metabolization of oxygenated compounds (methanol, acetone, methyl ethyl ketone, methyl isobutyl ketone, ethyl acetate, and butyl acetate) takes place followed by aromatic (toluene, ethylbenzene, and *p*-xylene) and halogenated compounds (dichloromethane and 1,2-dichloroethane). The series was observed due to the initial degradation of simple compounds by microbes. Furthermore, heterotrophic microbes from various communities develop the colonies in the biofilter beds. Two hypotheses are considered in the microorganisms’ colonization. First, the competition between microbial communities in the degradation of different compounds. The second one is the competition between substrates, which degrades easily biodegradable compounds. In this work, the degradation of toluene is higher than xylene. Due to the less solubility of xylene, the mass transfer rate was lower than the toluene to reach the biofilm. Also, steric hindrance caused by the presence of methyl group (CH_3_) and molecular mass of xylene supports in less biodegradation. Also, the authors suggest the stratification of microorganisms into colonies. For example, in the elimination of ethyl acetate and toluene, first centimeters of column degrade the ethyl acetate and the second column eliminates the toluene in the bioreactor. Such that it was understood the microbial colonies easily degrade the first preferred substance. Thus, different colonies of microbes degrade the various complex compounds present in polluted air/gas streams with 100% removal efficiency. The microorganism population was improved by considering the competition that exists amidst bacterial colonies and substrates [159].

**Figure 7.**
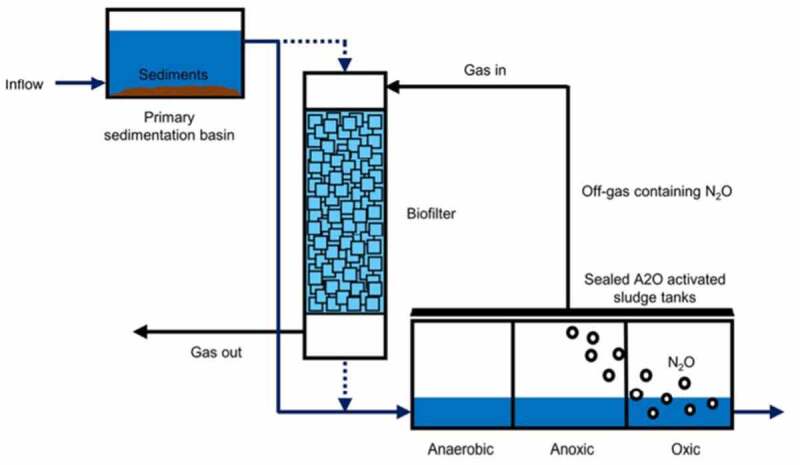
The schematic representation of biofiltration system for the removal of low concentration nitrogen dioxide emitted from wastewater treatment plants [163].

Biomass accumulation and crossing the standard level were controlled by the use of an agitator. When the biofilter bed reaches the pressure drop of 50 mm H_2_O/m the agitator automatically starts. By utilizing the shearing force, the clogged biomass from the filter medium was removed. Circulating pump and water spray were able to wash out the biomass into a water bath with freshwater. Irrespective of inlet concentration, the removal efficiency rate of all pollutants from this anti-clogging biofiltration (polyurethane bed) were the same for all the gases (ammonia (NH_3_) and volatile organic compounds ejected from food waste compost) equals 97%. Anticlogging biofilter with agitator produces stable performance and a potential candidate to produce efficient removal of VOCs [160]. Controlling emitted odor from the dead animals is one of the related topics to be observed. In this situation, biofilters are used in the removal of volatile organic compounds and ammonia along with controlling odor from dead pig and compost [160]. This process removed 37 volatile organic compounds. The transfer rate of pollutants was increased by elevating the inlet concentrations. It is worth noting that some volatile organic compounds at high inlet concentration inhibit microbial growth leading to a drop in removal efficiency. Nitrification in the biofilter retains the neutral pH (~7) of packing materials throughout the degradation of pollutants. No start-up time is required for the removal of ammonia due to high nitrification activity in the biofilter. In this work, removal efficiency percentages were found to be 79.2–95.4% for dimethyl sulfide, 81.9–94.0% for dimethyl disulfide, 76.7–99.1% for dimethyl trisulfide, and 92.9–100% trimethylamine at EBRT of 60 s. Thus, the empty bed retention time of 60 s was suitable for the removal of different VOCs using biofilters.

In this suggested regenerative biofilter model, the cons of regular/traditional technologies were reduced by isolating the microbes. It is used for the removal of formaldehyde [161]. For this enhanced version of biofilter, golden pothos (money plant) was chosen, and microbes are obtained from pebbles and roots of plants. Interaction between microbes and plants has to be studied in the removal of pollutants. For this analysis, different bacterial strains were utilized to understand the plant microbial interactions in the development of model biofilters with good efficiency in the removal of VOCs. *Arthrobacter aurescens*, *Arthrobacter oxydans*, *Bacillus subtilis*, *Bacillus cereus*, *Leifsonia xyli* and *Pseudomonas putida* are identified in this study from bacterial strains. This study helps to develop efficient biofilters with a better understanding of symbiotic microbes and plant interactions. So ornamental plants apart from providing beauty also provide regenerative bio-based indoor air clean with more efficient microorganisms [161]. However, each microorganism needs to be analyzed separately about its mechanism. Here, enzymatic degradation of formaldehyde was done in addition to physical adsorption. The result shows that *Arthrobacter aurescens* strains isolated from the plant had shown formaldehyde removal efficiency of 86.2% for interaction time of 24 h and initial concentration of 11.84 ppm.

**Figure 8.**
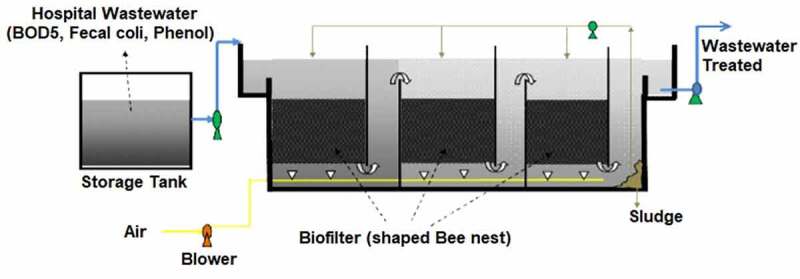
Schematic picture of aerated fixed film biofilter reactor in the treatment of hospital wastewater [162].

Aerated fixed-film biofilter setup was engaged in the treatment of wastewater expelled out from hospitals [162] (Figure 8). Major pollutants found in hospital wastewater are phenol, faecal coli, BOD and COD. This aerated fixed film biofilter consists of several bee nest filters with specific surface area ranging from 150 to 240 m^2^/m^3^. Endogenous bacterial colonies Bacillus Sp1, Bacillus Sp2, *Pseudomonas capica* and *Pseudomonas diminuta* are grown in the plastic bee nest filters. Wastewater from hospitals was given as an influent into these filters continuously. Some of the outlet effluent (treated wastewater) was fed again to the biofilter to support the growth of microorganisms. Performance of biofilter enhanced with suitable microorganisms, hydraulic residence time, and contact time (between microorganisms and pollutants). Decrease in hydraulic residence time has produced a reduction in the degradation efficiency. This aerated biofilter was found to produce highest elimination of BOD5 (96%), faecal coli (85%) and phenol (63%) respective at optimal time of 96 h. Another important substance nitrous oxide even at a low concentration (<200 ppmv) present in wastewater was removed with biofilter [163]. For this, a self-sustained biofilter setup was implemented which utilizes gravitational energy, gas flow, and a pressure differential of liquid medium (Figure 7). Two conditions were adopted for nitrous oxide removal in the presence of nitrogen gas and air. Raw wastewater is fed to biofilter continuously which acts as a source of nutrients and electron donors. Fed of synthetic wastewater with a background of nitrogen and airflow rate of 2,000 and 200 mL·min^–1^ was performed. The removal efficiency of nitrous oxide of 99% and >50% was obtained for nitrogen and air background, respectively. The process of nitrous oxide removal was supported by bacteria *Bosea* (2.39%), *Pseudomonas* (4.26%), and *Flavobacterium* (5.92%) [163]. Here, the technology was self-sustaining because of the direct gravitation force and differential in pressure, which transfer the liquid and gas into the biofiltration system. Hence, the external energy requirement will be less or no energy needed to transfer the influent into the system. The present study could be entirely adaptable after understanding its technical feasibility at the commercial level. For this, nitrogen dioxide concentration and fluctuation, temperature, and other toxic compounds have to be considered.

**Figure 9.**
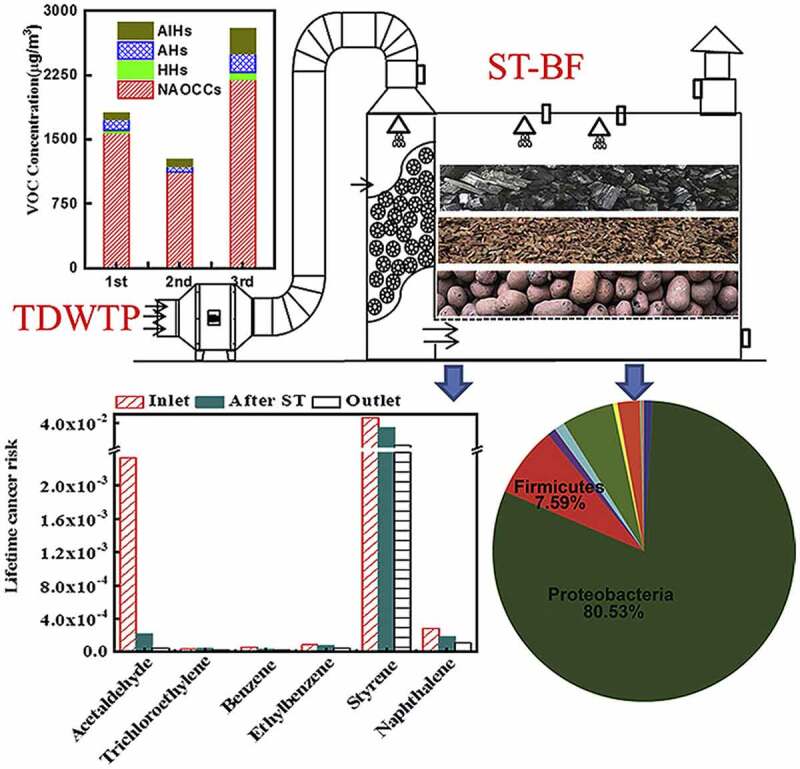
Schematic illustration representing spray tower combined biofilter in the removal of volatile organic compounds (VOCs) present in textile dye wastewater treatment plant, which reduces the risk of respiration diseases [164].

Biofilter combined with spray tower method was utilized in textile dye water treatment plant (Figure 9). This method ensures the reduced health risk by volatile organic compounds such as aliphatic, aromatic, and halogenated hydrocarbons along with compounds of nitrogen and oxygen. Each microbial genus has a significant effect on the removal of VOCs. Bacteria genus namely *Metallibacterium* was played a major role in the degradation of carbon disulfide and hydrogen sulfide from outlet gas. *Acidithiobacillus* had removed the nitrogen and oxygen compounds. Mainly acetaldehyde and benzene were removed with cancer and non-cancer volatile organic compounds by spray tower-biofilters. The growth or increase in bacteria from day 1 to day 90 represents the metabolism of volatile organic compounds, which shows the effective performance of biofilters. There are 50 types of volatile organic compounds with the concentration of 1.26 –2.79 mg/m^3^. The removal efficiency of volatile organic compounds was greatly enhanced from day 1 (38.1%) to day 90 (83.2%) by proteobacteria with ether lipid metabolism a dominant phylum present in biofilter (Figure 9). The mechanism involved was that VOCs released from textile dyeing wastewater treatment plant and microbial strain’s function were consistent for spray tower set up to promote the microbial growth. Depending on the pollutant variation in the microbial community was seen at the phyla/genus level. The spray tower biofilter was used for end of pipe treatment to meet the standard values provided by regulatory bodies. From this, complex VOCs (carcinogens or non-carcinogens) emitted from spray tower biofilter after treatment process confirms the significantly reduced ecological and health risks [164].

Upflow anaerobic sludge blanket coupled with a sequencing batch reactor was used to process the garlic wastewater. Individual run of upflow anaerobic sludge blanket and sequencing batch reactor under optimal conditions had shown chemical oxygen demand removal rate of 45% and 96% for 64 and 60 days, respectively. Proteobacteria of type α and type β was abundant in the sequencing batch reactor with sludge. These bacteria are responsible for the removal of phosphorous and nitrogen. Whereas after the coupling of two techniques the removal rate of chemical oxygen demand was 99% with 9800 mg/L of chemical oxygen demand influent. This shows the excellent coupling to treat garlic wastewater of high concentration [165]. Once again from this study, it was observed that the microorganisms played a potential role in the removal of phosphorous, nitrogen, and organic matters from wastewater. Also, the upflow anaerobic sludge blanket with sequencing batch reactor exhibits immediate start-up to treat high concentration of garlic wastewater treatment.

On the other hand, useful products were obtained from wastewater. Suitable filamentous fungi were utilized for bio-treatment of olive oil mill wastewater to achieve protein from it and also, reduction in the chemical oxygen demand [166]. In detail, wastewater from the olive oil mill acted as a source of high protein content microbial biomass. The biomass was bio-treated with the growth of filamentous fungi like *Aspergillus oryzae*, *Rhizopus delmar*, and *Neurospora intermedia* to get useful nutrients. The bio-treatment was supported by the addition of sodium nitrate as a source of nitrogen. After the inclusion of nitrogen, the biomass protein content increases as the dilution of the medium occurs with the decrease in cultivation time (96–48 h). For biomass concentration of 8.43 g/L, initial protein content was 15.9% which was increased to 29.5% and 44.9% before and after olive oil biomass dilution by nitrogen as the highest reported data. However, the remaining wastewater consists of high chemical oxygen demand after the fungal cultivation process and separation of biomass. Further, research on downstream processing of fungal cultivation has to be analyzed to support the next process like active sludge technique, etc. Same bioprocessing could be checked for the treatment of value-added wastewater streams composed of cell mass concentration, phenol-rich stream, etc. So, various toxic contaminants are converted into value-added products. Similar work was done in the production of edible protein from wastewater of wheat starch plant with fungi [167], in which the microbes, *Aspergillus oryzae* and *Rhizopus oryzae*, convert the organic substance into edible protein used as animal feed. This could be done by the consumption of sugar and hydrolyzing the starch a long chain of carbohydrates in wastewater. Thus, wastewater containing carbohydrates is converted into useful protein products by bio-treatment with fungi.

The volatile fatty acids find their role as ruminants that were used in the denitrification of the wastewater treatment plants. Excess sewage sludge and food waste slurry were utilized as sources to get volatile fatty acids with immersed membrane bioreactor (iMBR) [168]. Such volatile fatty acids obtained from retrofitted immersed bioreactor were found to be the best source of bio-based carbon. This was substituted instead of conventional fossil fuel of methanol by carrying out the denitrification in the wastewater treatment plant. Through experiments, the accumulation of volatile fatty acid was monitored for thermal and sodium hydroxide treatments and no pretreatment. Pretreatment of excess sewage sludge had not produced any significant improvement in the accumulation of volatile fatty acids even at a pH of 5. For pH 12, the total volatile fatty acid was recorded to be 13.99 g/L from excess sewage sludge. The thermochemically pretreated food waste slurry had accumulated more volatile fatty acid at various pH than the thermally pretreated food waste slurry. Also, the pretreatment with sodium hydroxide, the bioreactor substrates of excess sewage sludge, and food waste slurry had increased their chemical oxygen demand, at lower exposure time and temperature. Finally, the accumulation of volatile fatty acid varies for food waste slurry as substrate whereas no effect with a substrate of excess sewage sludge [168]. Thus, the study shows the importance of substrate determines the accretion of volatile fatty acids. In conclusion, it was observed that the performance of the biofilters depends on the contaminant loading, i.e., loading rates, microbial growth, filter media, pH, temperature, nature of the substrates, etc. It is necessary to have a clear knowledge of the contaminants to be removed and the effect of microorganisms.

### 4.3 Bioscrubber

In the 1970s, German have implemented bioscrubbers to treat the volatile organic compounds present in waste gas ejected from wastewater treatment plants [169,170] (Figure 3c). The process was carried out with the bioscrubber filtration setup. The bioscrubber consists of two main parts- the absorption column and the bioreactor unit (activated sludge reactor) [171]. The absorption unit is generally constructed with plastic materials that offer high specific surface area and high porosity. This configuration supports the effective mass transfer of contaminants and avoids biofilm clogging, respectively [171]. The gaseous pollutants are transferred into an aqueous solution (aerosol) present in the absorption column [169]. Followingly, he dissolved polluted compounds in the aqueous phase are passed into a bioreactor for regeneration. Mostly, the bioreactor is a tank of a large volume than the absorption column and contains activated sludge. The wastewater contaminants are aerobically biodegraded in the activated sludge unit by the microorganisms [171]. Treated water from the bioreactor was sent back to the top of the absorption column. Gas and aqueous phase are allowed to circulate in co or co-counter current direction in the absorption column. In this stage, highly soluble pollutants are removed [15]. The aqueous phase should be supplied with the nutrients to confirm the better growth of microorganisms in turn supporting biodegradation of pollutants (VOCs). Optimum performance of the absorption unit was attained with the control pH of the aqueous phase by suitable acid or alkali titrants [169]. The control over pH of aqueous phase exists the highly water-soluble chemical substance such as alcohols, fatty acids, hydrogen sulfide, sulfur dioxide, and aldehydes are easily removed [172]. The performance of bioscrubber was enhanced by including emulsifiers such as phthalate and silicon oil. Emulsifying agents support the mass transfer of VOCs from the gas phase to the aqueous phase. Thus, transferred contaminants of less solubility are removed efficiently [15]. Bioscrubber-based wastewater treatment solutions offered by US-based company has employed thiobacillus bacteria for the efficient conversion of hydrogen sulfide [173]. Depending on the specific need, the thiobacillus bacteria was used in the conversion of hydrogen sulfide and odor from waste gas into sulfate and sulfide. Bacterial growth was assured by the available micronutrients and solution mixture of nitrogen, phosphorous, and potassium [173].

The merits of bioscrubber, when compared to other biofilters, are as follows [15,169]:

1. Stable operation.
2. Control of pH and microorganism growth.
3. No pressure drops and clogging of packing materials in the absorption column.
4. Small space is enough for the bioscrubber setup.
5. Aqueous phase has a low toxic concentration.
6. Reliable technique and results are predictable.

The demerits of bioscrubber include [15,169] are as follows:

1. Operational cost is high with the complicated initial procedures.
2. Production of more sludge and its disposal.
3. Bioscrubber is cost-effective for the soluble pollutants (VOCs) with Henry’s coefficient less than 0.01 and for the gaseous pollutants <5 g m^−3^.
4. Bioscrubber had produced higher efficiency equals 98%.
5. Require stagnation time to start up the next treatment.
6. Wastewater is produced.
7. Not applicable to pollutants of low aqueous solubility and highly volatile.

Despite merits of bioscrubber given above, the real-time employment of bioscrubber in the removal of VOCs remains less. A mixture of aromatic, oxygenated, and chlorinated compounds (VOCs) is treated by bioscrubber, with an automizing absorbing column process. The removal efficiency of VOCs remained to be 35%, which was very less. For oxygenated compounds, it was found to be 55–80%. To achieve high efficiency, mass transfer of compounds from gas to aqueous phase and optimization of automized column (hydrodynamic property) need to be analyzed to scale up the activity [174]. Hence, it is necessary to develop bioscrubbers by optimizing the relevant parameters to consider the technique for its potential role in the removal of VOCs [175].

## 5. Biofiltration technique in the removal of heavy metals

As said, heavy metals released from industrial and traditional activities pollute the water resources and soil. Hence, it is necessary to remove those metal contaminants. Conventional technologies are available in wastewater treatment. The conventional technologies were limited in their performance in the accumulation and discharge of heavy metals and the detoxification process. Thus, it failed to achieve the permitted ppm concentration of heavy metal present in water and land resources. AltMoreover, the shifting of industries to rural areas had not provided the complete solution. Still, contamination of water and land resources by heavy metal ion disposal exists [176]. However, green technologies were considered for several merits, especially eco-friendly safeguarding natural environment by removing heavy metals. This section provides phytoremediation (botanical biofilters), microorganism-based biofilters, and biomimetic membranes for the removal of heavy metals. The various biological processes engaged in the removal of heavy metals are provided in Table 4, Table 5.

**Table 4.** **Key processes adopted in the treatment of polluted stormwater** [184]

Pollutant in stormwater	Key processes
Sediment	Physical filtration is done by filter media Settlement during ponding
Nitrogen	Nitrification and denitrification Decomposition Adsorption Biotic assimilation by plants and microbes Physical filtration of sediment-bound fraction
Phosphorous	Decomposition Adsorption Biotic assimilation by plants and microbes Physical filtration of sediment-bound fraction
Heavy metals	Oxidation and reduction reactions Biotic assimilation by plants and microbes Physical filtration of sediment-bound fraction
Pathogens	Adsorption and desorption Physical filtration by filter media Natural or predation die-off
Organic micropollutants	Adsorption Biodegradation

**Table 5.** Various biological-based processes employed in the removal of heavy metals in the treatment of water

Heavy metals	Methods	Sources	Governed mechanism	References
Ni, Cd, Pb, Cr, Hg, and Co	Bioaccumulation	*Azolla filiculoides*	Phytoaccumulation	[197]
Fe, Pb, and Zn	Bioaccumulation	*Azolla pinnata* and *Lemna minor*	Phytoaccumulation	[198]
Pb(II), Cd(II), and Cr(VI)	Bioadsorption	Methane-oxidizing epipelon	Biofilm	[199]
Cr	Bioadsorption	*Pseudomonas koreensis*	Bioremediations	[200]
As	Bioadsorption	Biochar from rice straw	Electrostatic attraction, ion-exchange, and π–π/n-π interactions	[201]
Cd and Pb	Bioadsorption	Biochar from peanut shell	Freundlich isotherm model	[202]
Cu, Cd, Cr, Ni, Fe, Pb, and Zn	Bioadsorption	*Phragmites australis* and *Typha latifolia*	Retention	[203]
Cd, Pb, Ni, Zn, Cu, and As	Bioadsorption	Biochar from tree, weed, and crop	Adsorption	[204]
Cu(II) and Cd(II)	Bioadsorption	Micro-algae/bacterial biomass	Langmuir model	[205]
Cu(II), Cd(II), and Pb(II)	Biosorption	Biochar from vegetable biomass	Electrostatic attraction	[206]
Cu, Pb, and Zn	Biosorption	Beetroot fibers	Retention	[207]
Pb(II), Cd(II), Cu(II), and Ni(II),	Biosorption	Coco-peat biomass	Langmuir and Freundlich isotherm	[208]
Cd(II)	Biosorption	Biofilms from biotrickling filters	Langmuir isotherm model	[209]
Pb(II)	Biosorption	Cottonwood	Precipitation, electrostatic outer- and inner-sphere complexation	[210]
Pb(II)	Biosorption	Olive pips	Biosorption	[266]
Cu and Zn	Biosorption	*Synechocystis* sp., *Chlorella* sp. and *Scenedesmus* sp.	Autoclave	[211]
Cu and Pb	Biosorption	Chara algae	Best pH	[212]
Cd, Pb, and Ni	Biosorption	*Nitzschia palea* and *Navicula incerta*	Filtration	[213]
Cd (II) and Ni (II)	Biosorption	*Cymodocea nodosa*	Langmuir isotherms	[214]
Cd(II) and Pb(II)	Biosorption	*Pleurotus ostreatus*	Immobilization	[215]
Ni(II)	Biosorption	*Lycopersicum esculentum*	Batch method	[216]
As(III)	Biofilm	Biochar and Periphytic biofilm	Pseudo-second-kinetic model	[217]
Cd	Biofiltration	*Lamellidens marginalis*	Bioaccumulation	[218]
Fe, Mn, and NH_3_-N	Biofiltration	Oxidizing bacteria	Chemical oxygen oxidation; water redox environment	[219]
Cr(VI)	Biofiltration	*Pseudomonas taiwanensis*	Michaelis–Menten kinetic model; Ottengraf-Van den Oever model	[220]
Pb and Cd	Biofiltration	*Pistia stratiotes* L., *Salvinia auriculata* Aubl., *Salvinia minima* Baker, and *Azolla filiculoides*	Rhizofiltration	[221]
Fe(II), Mn(II), and As(III)	Biofiltration	*Gallionella, Leptothrix, Pseudomonas, Hyphomicrobium, Arthrobacter, Alcaligenes*	Denaturing gradient gel electrophoresis (DGGE)	[222]
As(III)	Biofiltration	Burkholderiaceae, Comamonadaceae, Rhodobacteraceae, and Xanthomonadaceae	Autotrophy; Heterotrophic oxidation	[223]
Pb and Cd	Biofiltration	Polylactic acid – fish scale extracted hydroxyapatite (HAp)	Ion exchange, dissolution, and precipitation on HAp	[188]
Zn and Pb	Biofiltration	Stormwater	Bioretention	[224]
Pb(II)	Biofiltration	*Furcraea andina*	Biofilm-forming bacterium	[225]
Cd, Cr, Co, Ni, and Pb	Biofiltration	*Eichhornia crassipes*, *Lemna minor* and *Azolla pinnat*	Bioaccumulation	[226]
Cr, Co, Fe, Mn, Pb, and Zn	Biofiltration	*Eichhornia crassipes*	Bioaccumulation	[227]
Pb, Cd, Zn, Cu, As, and Cr	Biofiltration	*Taxiphyllum Barbieri*	Phytofiltration	[228]
Ni(II) and Co(II)	Biofiltration	*Escherichia coli*	Biofilm formation	[229]
Ni, Cd, Cr, Fe, Pb, and Cu	Biofiltration	*Pseudokirchneriella subcapitata*	Bioremoval	[230]
Pb	Biofiltration	Leuconostoc mesentroides and Lactobacillus case	Biofilm	[231]
Cr(VI)	Biofiltration	Cellulose; *Eichhornia crassipes*	Langmuir isotherms	[232]
As and Hg	Biofiltration	Activated coconut shell	Biosorption	[233]
As	Bio-oxidation/adsorptive filtration method	Acidothiobacillus ferrooxidans	Filtration and adsorption	[234]
As(II), Ca(II), Mg(II), Ni(II), and Zn(II)	Biopolymer ion exchange	Organic polymer pectin hybrid	Ion exchange	[235]
Zn (II), Pb (II), Cr (III) and Cr (VI)	Biopolymer filtration	Starch	Ultrafiltration	[236]
Cr(VI)	Biopolymer-sorption	Biocomposite beads (alginate)	Sorption process	[237]
Cr(III), Pb(II), and As(V)	Biopolymer-sorption	Carboxymethyl cellulose and alginate-based hybrid	Sorption process	[238]
Pb, Cd, Cu, and Zn	Bioremediation	*Serratia rubidaea* NCTC12971	Microbial detoxification	[239]
Cd, Pb, and Ni	Bioremediation	*Phragmites australis*	Phytoremediation	[240]
Fe, Cu, Pb, Cd, and Zn	Bioremediation	*Cyperus rotundus*	Phytoremediation	[241]
Tl, Cd, Zn, and Pb	Bioremediation	*Callitriche cophocarpa*	Phytoremediation	[242]
As, Cd, and Hg	Bioremediation	*Eichhornia crassipes*	Phytoremediation	[243]

### 5.1 Macrophyte biofilters

Several macrophytes (aquatic plants) growing in ponds and streams possess the property of capturing the heavy metal contaminants present in waterbodies and accumulating in various parts of them. Although aquatic plants require heavy metals for their growth, a higher concentration of heavy metals in water resources produces toxic effects on the entire living creatures. Macrophytes act as a natural filter (biofilter) by capturing the heavy metals from polluted water sources. Bioaccumulation and biomagnifications of heavy metallic ions in the macrophytes lead to the biofiltration of contaminated water. The processes of absorption of heavy metals by botanical species are known to be bioaccumulation and biosorption. The absorption property of aquatic plants depends upon the affinity for heavy metals. Accumulates the absorbed metal ion in various parts of it. The process is simple and cost-effective. *Azolla* fern had shown passive absorption of heavy metals from contaminated water [176]. These ferns have exhibited strong storage and metal-binding capabilities due to their high attraction for bivalent metal ions. Then, the heavy metal bound into *Azolla* fern was safely discarded to avoid its interaction with air and water sources. *Azolla* plants hold copper and cadmium of 3–5%, chromium 5–11%, and lead 5–12%. The biomass *Azolla* with heavy metals is heated at a very high temperature (incineration process) to convert into ash and acid wash of the biofilter. The acid wash makes the biofilter for its reuse [177]. Similarly, *Azolla caroliniana* was employed in the removal of mercury and chromium ions from municipal water [176]. Apart from heavy metal ion capture, the plants can also be used in soils and water contaminated with radioactive substances, chemicals, organic solvents, chemical fertilizers, pesticides, etc. A cultured solution consisting of mercury and chromium ions was treated with *Azolla caroliniana*. The tissue of *Azolla caroliniana* had shown the highest capture of chromium (III) in the range 200–48,000 mg/dm^3^ and for mercury, the absorption was up to 578 mg/dm^3^. These works demonstrate the use of fern plants as bioaccumulator engaged in the efficient removal of heavy metals [176,177]. Thus, the phytoremediation process was facile to carry out the heavy metal capture in an eco-friendly way. Heavy metals are collected in the tissues or roots of plants and decomposed harmlessly. Hence, phytoremediation techniques utilize botanical creatures to store a high quantity of heavy metals.

Beetroot fibers were employed in the biosorption process in the treatment of hard water treatment and desalination of water [178]. Beet fiber had shown a low retention capacity for nickel, whereas high for lead at the optimal pH ~6.6. Further, total dissolved solids were declined in their level from hard water and seawater [178]. Absorbed heavy metals from beetroot fibers can be removed by changing the pH, which produces the destruction of active metal–ligand form. Hence, the metal from aquatic biomass was removed successfully. Merits of beetroot fiber biofilters include low cost, operate a wide range of pH and temperature, are efficient in the treatment of water salinity and hardness, and also remove low concentration metal contaminants. Aquatic plant *Eichhornia crassipes* had shown great affinity for heavy metals like copper (Cu), chromium (Cr), lead (Pb), manganese (Mn), and zinc (Zn) [179]. These plant parts have shown a higher presence of humic acid, which is found to be the reason for metal binding and water retention capacity. Phragmites australis and *Typha latifolia* have shown synergetic effects in acquiring heavy metals from contaminated water [180]. The statistical analysis shows the significance value p < 0.001 after 15 days of retention time. The result shows that 15 days of treatment is enough for the highest removal of metal contaminants. Iron was accumulated higher followed by zinc (1,034.2), lead (113.2), chromium (48.4), nickel (20.0), and cadmium (21.6) by the whole plant species in terms of mg/kg [180]. Bioaccumulation and natural biodegradation of heavy metals by utilizing macrophytes is a suitable sustainable technique for the treatment of wastewater. This method suggests the complete removal of heavy metals by plant species. Also, the work proves that the greens grown from polluted water may affect its consumers.

### 5.2 Stormwater biofilters

**Figure 10.**
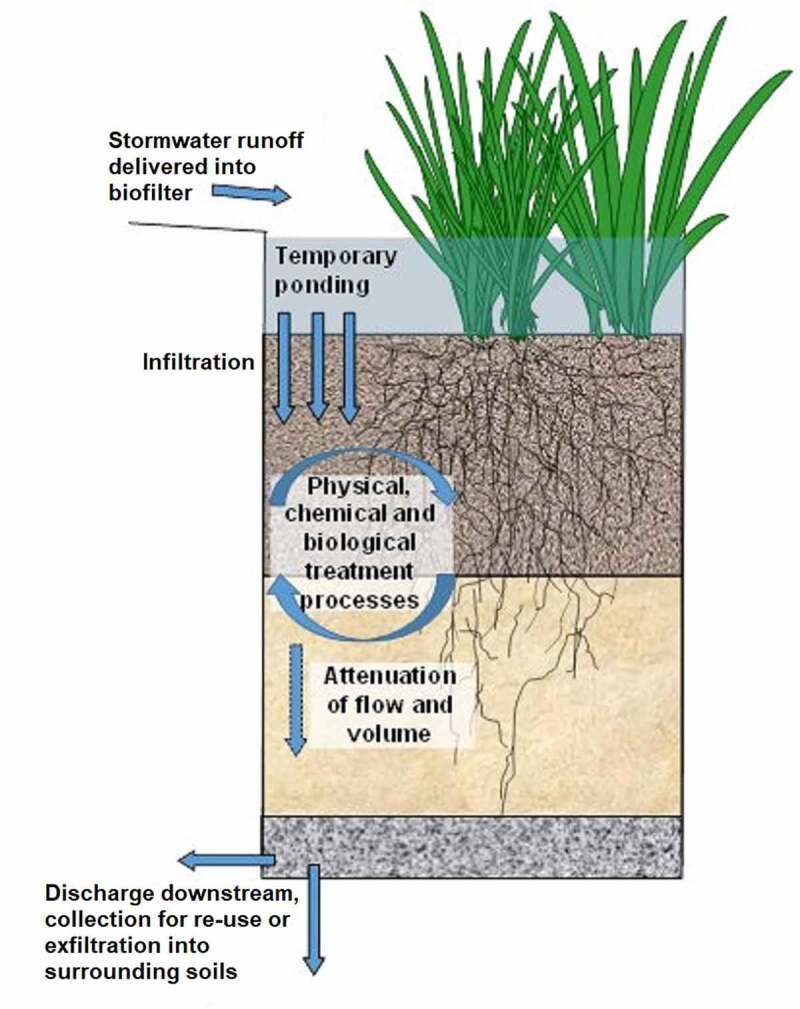
Typical stormwater biofilter working model [184].

In addition to aquatic biofilters, there exist biofilters to treat stormwater in the removal of heavy metals and other contaminants. The stormwater biofiltration could be designed and optimized based on the requirement. The synergic effect of the plant, microbes, and filters in the stormwater filter setup plays a significant role in the removal of contaminants from wastewater. Figure 10 shows the typical stormwater biofilter setup. Table 4 provides the processed steps in the removal of various contaminants present in stormwater. Removal of copper, cadmium, lead, and zinc was studied with a stormwater biofiltration system [181,182]. Conventional storm biofilters have the potential in capturing heavy metals up to ~90%. The design got modified with a topsoil layer of 100 mm thickness. Due to the enhanced sorption capacity of the topsoil layer, the metals were retained in it. Investigations were done at the temperatures of 2°C, 8°C, and 20°C. Excluding copper, other metal removals were not affected by the temperature. The efficiency of removal of copper got increased with a decrease in the temperature, i.e., enhancement in biological activity at a lower temperature. This effect had expressed the elevated ejection of copper along with organic matter decomposition. This study shows that temperature acts as an important parameter in the adsorption of heavy metals by biofilters [181].

**Figure 11.**
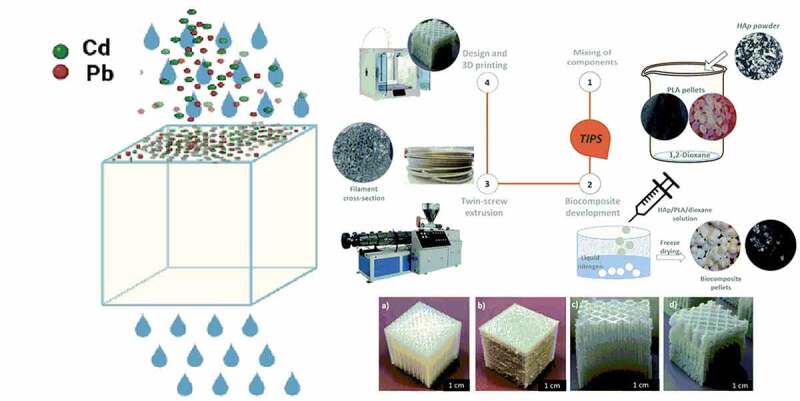
Diagram representing the 3D-printed monolithic biofilters based on a polylactic acid (PLA) – hydroxyapatite (HAp) biocomposite for heavy metal removal from an aqueous medium. (a) Reference PLA filter with a uniform porosity. (b) Corresponding PLA/Hap filter. (c) Reference PLA filter with gradient porosity. (d) PLA/Hap filter [188].

Following, another work was performed on biofilters for the removal of heavy metals from stormwater [182]. In this analysis, various parameters during biofiltration operation (for 8 months) were taken into account to determine the performance of the biofilter. Various parameters are taken such as type of filter media, depth of filter media, vegetation, the concentration of the pollutant, operational time, and flow rate. Amidst these parameters vegetation and filter, media type plays a major part in the removal of metal. Outflow concentration of the metal increased with the large depth of biofilter media. This was due to the mobilization and leaching effect of the metals. In the removal of iron, 4% of the catchment area had to be taken as a biofilter area with enriched organic content. The treatment efficiency rate of iron was better than the lead and copper. This stormwater harvesting biofilter had shown satisfactory removal of all metals (except aluminium and iron) which meet a standard measure of potable water. The same water quality was found to be applicable for irrigations and drinking purposes [182] Figure 11.

Stormwater biofiltration performance was evaluated in the removal of heavy metal. The machine learning programs like neural networks, multilinear regression, and random forest were used for the analysis [183]. The consistent employability of biofilters in pollution control was aided by this machine learning approach. Both physical design and operational parameters (here pollutant concentration, flow rate) of biofilter contribute to the removal of heavy metal. Nash–Sutcliffe Efficiency (NSE) median values are 0.995 (Cd), 0.317 (Cr), 0.762 (Cu), 0.636 (Fe), 0.726 (Ni), 0.896 (Pb) and 0.656 (Zn) from random forest. These results from the random forest were more efficient than the neural network and multilinear regression. Determining the risk quotient value (RQ < 1) from the outflow concentration could help optimize the standard quality of water [183]. Apart from heavy metal capture, the other ecosystem process of stormwater biofilters are aesthetics, pollinator habitat, potential water supply, and sequestration. If underlying soils have high infiltration capacity then the biofilters recharge by using the groundwater. Further studies are to be carried out to evaluate the capacity of stormwater biofilters in the removal of other pollutants such as synthetic chemicals, pathogens, organic contaminants, etc. In the removal of nutrients, the biofilter medium of low organic content must be utilized to reach the standard drinking quality of water. Further, the protection of the aquatic ecosystem should be considered while carrying out the biofiltration process.

### 5.3 Microorganism-based biofilters

Heavy metals are degraded, transformed, or reduced into less or no toxic contaminants through microbes are known to be bioremediation by microorganisms [185]. Also, the microorganisms could be employed in controlling the odor during biofiltration. Bacteria, fungi, algae, and plants were used for bioremediation. Mostly, the bacterial enzyme was applied for degradation through the metabolization process [186]. Several biological and environmental factors affect the growth of microbes. In the biological factors, the microbe’s enzyme activity, size, composition, mutation, and horizontal gene transfer play a key role. Environmental parameters like temperature, pH, nutrients, moisture, and oxygen concentration play a crucial role [185,186]. To overcome these obstructions, genetic variation was executed for the microbes [187]. Microbes after genetic variations possess resistance toward the above-included factors. So, bioremediation was performed completely by the modified microorganisms. Bacterias used for bioremediation are *Alcaligenes*, *Bacillus*, *Citrobacter*, *Escherichia*, *Klebsiella*, *Pseudomonas*, *Rhodococcus*, and *Staphylococcus*. In fungi, *Aspergillus*, *Penicillium*, *Pleurotus*, *Rhizopus*, and *Saccharomyces* are commonly used for bioremediation.

From a multi-component system, heavy metals are removed by sulfate-reducing bacteria. The removal efficiencies are appreciable for both low and high metal concentrations. At higher concentrations of metal, the removal efficiencies were decreased. This was due to inhibition of sulfate and chemical oxygen demand reduction [189]. Chemically modified *Penicillium chrysogenum*’s biomass (Mycan) finds its potential in the removal of arsenates [190]. A cationic polyelectrolyte and surfactants like dodecylamine and hexadecyl-trimethylammonium bromide are used for the modification of fungus biomass. In this study with Mycan, the modification of biomass has increased the total ionic content of biomass (0.57 meq/g). The biomass modification can be done with easy steps and low cost. Heavy metal biosorption results for Mycan-modified hexadecyltrimethylammonium bromide was 37.85 mg As/g, whereas dodecylamine and cationic polyelectrolyte produced 33.31 and 56.07 mg As/g with a pH value of 3 [190]. Even a low concentration of arsenate could be removed by this modified biomass. Uranium bioprecipitation was made possible with the *Deinococcus radiodurans* known to be polyextremophile bacterium resistant to radiation. Due to lyophilization, it holds non-specific acid phosphatase activity supporting precipitation of uranium. Hence, *Deinococcus radiodurans* can be employed in the capture of heavy metals including cadmium [191, 192–194]. Further, these bacteriophages possess the ability to treat the bulking of sludge, dye, and foaming in wastewater [195–197].

Vertical flow biofilter of laboratory-scale conditioned with sulfate-reducing bacteria (SRB) was used to remove the lead and zinc, in the treatment of rainwater [195]. The removal efficiency was calculated by taking into account the concentration of lead and zinc along with the carbon-to-sulfate ratio. Maximum removal of heavy metals 80% was observed at a 1:1 ratio of carbon and sulfate for 126 days of treatment [195]. Very recent microalgae diatoms with silica shelled of eukaryotic cells were found its capacitance in the sensing of different pollutants present in wastewater [196]. The microalgae act in two ways with the perspective of nanotechnology: first to detect the pollutants and next to act as smart nanocontainers. These microalgae are nanocontainers of eukaryotic unicellular microorganisms capturing and carrying even trace metals, drugs, dyes, hydrocarbons, polymers, etc., from wastewater. This was possible by the creation of bonding of nano-smart microalgae with the pollutants of choice of ligands. While compared to the artificial silica-based nanomaterials, the natural microalgae of nanosize are affordable in treating the pollutants [196]. So, microorganisms under suitable parameters could effectively capture heavy metals.

### 5.4 Biomimetic membranes

Researchers engaged in the fabrication of biological membranes for water filtration mimic the cellular membrane. These biological membranes are known as biomimetic membranes, which do water filtration as done in cellular membranes naturally for a billion years [244]. The structure and functions of the biomimetic membrane resemble the natural cellular membrane [245]. In the biological cells, water transportation takes place through the channels present in the cell membrane formed by the biomolecule known as aquaporin [94]. In 2003, Peter Agre was honored with the Nobel Prize for his deepest work on aquaporins’ water channel [246]. These are intrinsic proteins forming pores in the cell membrane. The pores are responsible for the effective transport of water alone through osmosis and avoid the entry of any other ionic species or solutes [247,248]. Aquaporins isolated from cell membranes are cultured more in the presence of microbes (bacteria, yeast, etc.). Subsequently, cultured aquaporins are fixed on the substrate of the polymer membrane. This polymer membrane was observed to be a matrix of lipids and proteins. Water or solute selection by the aquaporins depends on parameters such as size, hydrogen bonding, and electrostatic interactions. Positively charged ions were repelled from the surface of the cellular membrane by electrostatic repulsion. On the other hand, reorientation of water dipole in the aquaporin water channel supports the transport of individual water molecules [246,249,250].

Research works were demonstrated on the production of enhanced aquaporin biomimetic membranes with the support of recent engineering technologies. The electrokinetic method had generated a stable and uniform aquaporin functionalized membrane for water purification. The salt rejection rate of 97.8% with a water flux of 7.45 Lm^−2^h^−1^ was observed under forwarding osmosis with this membrane [251]. Recently, Zhao et al. reported on the optimization of governing parameters such as protein-to-lipid ratio, loading of proteoliposome, and cholesterol to yield aquaporin biomimetic thin-film composite membrane of good performance at low cost [252]. Increasing proteoliposome and optimal protein-to-lipid ratio with cholesterol better salt rejection rate and water flux were achieved. Now, commercial aquaporin membranes are available in the market for reverse osmosis and forward osmosis type of applications [253, 254, 255]. These membranes are superior in the selection and transport of molecules in water purification systems than the conventional technologies.

**Figure 12.**
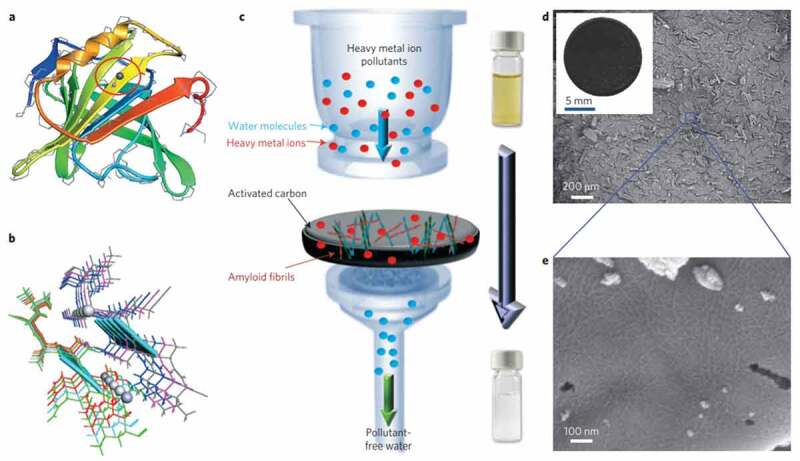
Schematic illustration of amyloid fibrils coupled with activated carbon membrane as an adsorber of heavy metal ions. (a) Structure of the β-lactoglobulin protein with the strongest heavy metal-binding motif highlighted, 121-cys, with a lead ion attached. (b) Amyloid-forming 121-cys-containing fragment (LACQCL) from β-lactoglobulin with docked Pb metal ions. (c) Schematic representation of heavy metal ion purification by amyloid–carbon adsorbers, and photographs of Na2PdCl4 solution changing color from yellow to colorless after filtration due to the adsorption of palladium heavy metal ion pollutants onto the composite membrane. (d) SEM image showing the surface of the composite membrane, with the visual aspect of the membrane shown in the inset. (e) Higher-magnification SEM image of the membrane, demonstrating the assembly of the amyloid fibrils onto the activated carbon surface [259].

Currently, protein-based amyloid fibril hybrid membranes were investigated for water purification by capturing heavy metal pollutants [257–259]. Amyloid fibril-based membranes have shown increased efficiency in acquiring metal ions when compared with those other methods like reverse osmosis and nanofiltration [256,257]. Whey protein a by-product of chess industries acts as a source to synthesis β-lactoglobulin. At low cost, amyloid fibril membranes are fabricated from this β-lactoglobulin with standard simple synthesis protocols [258]. Thus, produced membranes are suitable for large-scale water purification systems. A comparative study was performed between amyloid fibril from β-lactoglobulin and activated carbon in acquiring heavy metal ions. Relative specific adsorption capacity per filtration cycle (in µg mg^−1^) was higher for the β-lactoglobulin amyloid fibrils than the activated carbon engaged in the capture of gold, mercury, lead, and palladium ions [259]. This was due to effective binding sites available at β-lactoglobulin amyloid fibrils to capture different metal ions. Adsorption efficiency of 99.98%, 99.5%, 99.7%, and 99.84% for gold (Au), mercury (Hg), lead (Pb), and palladium (Pd) ions was reported. Enhanced adsorption efficiency wasdue to the synergic effect of the high metal ion capture property of amyloid fibrils and high porosity characteristics of activated carbon [259] (Figure 12). Previously, metal–ligand interactions of amyloid fibril had shown an efficiency of ~99.6% in acquiring arsenate and arsenite oxidation forms of arsenic (As) [257]. This adsorption technology proved to be cost-effective and efficient in the removal of arsenic. Delay in saturation of the membrane allows consecutive recycling [257,259]. In the other work, amyloid fibrils were used as a functional scaffold for templated metal-organic framework (ZIF-8) hybrid aerogels in universal water purification [260]. In the case of heavy metal ion removal, the porous nature of these hybrid aerogels and the presence of amino groups in the matrix of amyloid were found to be the reasons for the efficient scavenging of metal ions. Existence of valence force between the reactive binding sites of hybrid aerogels and metal ions, chemisorption takes place. In this experiment, mercury ions are scavenged with a high correlation coefficient of 0.9979 [260]. Recently, amyloid superstructures were found to be potential adsorbers of lead ions from an aqueous medium [261]. It is worth noting that captured metal ions were recovered easily from the saturated protein-based amyloid membranes and used for other applications. Amyloid fibril membranes are considered for their low cost and facile synthesis. Its efficiency in the treatment of industrial wastewater and water resources from various pollutants.

## 6. Key challenges and future perspective

The above biological-based filter technologies have shown great potential in the removal of volatile organic compounds and heavy metals in the treatment of wastewater. However, the biofiltration method has its limitations and challenges to be rectified.

- First, the microbial community and its growth have a very important role in the performance of biofilters. Research studies exist on microbial activity in the degradation of contaminants present in water. While carrying out the degradation activity, the idea of extending the same for mass-scale has to be taken into account. Many works are not adapted into real water treatment applications after their execution in the laboratory scale at the pilot-level approach.
- A clear picture is not known about the mechanism of metabolic pathways of microorganisms involved in the degradation of pollutants. It is unavoidable to see the sights of metabolic pathways of microbes for the entire removal of contaminants [119]. In turn, it depends on the nature of the microbe, suitable genus, and its community in the biofilter bed [119,185,187,189]. Hence, it is necessary to receive complete knowledge about microbial growth and degradation mechanisms to achieve the real employment of biofiltration technology.
- Next, when the concentrations of contaminants have increased, it leads to the growth of a thick biofilm layer. The adsorption takes place by the microbes on the surface of the biofilm. Also, it enters into the depth of the biofilm. After a period, the diffusion of contaminant to reach the depth of biofilm was stopped. Thus, giving rise to inactive microbial biofilm with varying pore sizes [11]. In this regard, multi microbial cultures can be employed to improve the removal of various pollutants with less degradation time [262].
- The contaminants were from different sources like industrial wastewater, municipal sewage, water treatment plants, petroleum refineries, pharmaceutical waste, etc. Biofiltration equipment or method has to be designed and optimized in a way by counting the characteristics and quantity of the pollutants present in wastewater. Also, the treated water and acquired metals should be considered for safe disposal or recycling/valuable products.
- In the future, economically affordable biofilters with better technical design at low investment by addressing the above challenges will be done. This is possible with the intelligence of machines. Nowadays, artificial intelligence (AI) has provided its candidature in vast areas including water treatment [263–266]. Where it could predict the performance of various adsorbents (microbes) concerning different types and quantities of pollutants in wastewater [6,8,9]. Further, simultaneous removal of contaminants without any secondary pollutants and fouling effect, with value-added products will be expected [114,262]. View on to recent studies, it is possible to achieve such a required biological-based filtration by hybridization techniques for removal of pollutants from wastewater [109,220,240]. Hence, in the forthcoming days, it is possible to achieve the best water treatment biobased technique controlled by AI.

## Conclusion

This review article depicts the importance and requirement of biofilters in the treatment of wastewater to attain a sustainable clean environment. Microorganisms and botanical species are engaged in the biodegradation or capturing of pollutants present in wastewater. The performance of biofiltration relies on important parameters such as filter bed media, microorganisms, temperature, pH, moisture, pressure, and nutrient. Based on the pollutants, these parameters are optimized to obtain high removal efficiency. Biological-based filters, in which microbes and plants are the heart of the system, are utilized in the removal of different pollutants. The articles discussed have shown the performance of various types of biofilters for wastewater treatment. Further, from the review, it was very clear that deep understanding needed to come out with universal biofilters for all time. Such biofilters are expected to degrade/capture the highly toxic volatile organic compounds, heavy metals, and other toxic/non-toxic materials to maintain a clean and safe environment. All discussed research works emphasize the responsible role of initial level inlet concentration, loading rate, pH, retention time, temperature, microbial growth, flow rate, etc., in the complete degradation of pollutants. After understanding the pollutants, the flexible biofiltration setup has to be fabricated. Recently, the amyloid fibril membrane was greatly used in the innovative biofiltration construction along with activated carbons to achieve a sustainable universal water purification process. Now machine learning became the hope and recent trend for achieving betterment in the design and performance of varied technologies. Biofilter models supervised by artificial intelligence (AI) are able to predict the adsorption of different filtration models by various biological organisms. Relative parameters and conditions of experiments could be pre-planned before starting up of the real-time process. In the near future, biological-based filters under the control of AI will definitely provide universally adaptable and acceptable sustainable solutions to solve the problems caused by several pollutants.
